# Collagen-Like Proteins in Pathogenic *E. coli* Strains

**DOI:** 10.1371/journal.pone.0037872

**Published:** 2012-06-06

**Authors:** Neelanjana Ghosh, Thomas J. McKillop, Thomas A. Jowitt, Marjorie Howard, H. Davies-Strickleton, David F. Holmes, Ian S. Roberts, Jordi Bella

**Affiliations:** 1 Manchester Interdisciplinary Biocentre, University of Manchester, Manchester, United Kingdom; 2 Faculty of Life Sciences, University of Manchester, Manchester, United Kingdom; University of Massachusetts Medical School, United States of America

## Abstract

The genome sequences of enterohaemorrhagic *E. coli* O157:H7 strains show multiple open-reading frames with collagen-like sequences that are absent from the common laboratory strain K-12. These putative collagens are included in prophages embedded in O157:H7 genomes. These prophages carry numerous genes related to strain virulence and have been shown to be inducible and capable of disseminating virulence factors by horizontal gene transfer. We have cloned two collagen-like proteins from *E. coli* O157:H7 into a laboratory strain and analysed the structure and conformation of the recombinant proteins and several of their constituting domains by a variety of spectroscopic, biophysical, and electron microscopy techniques. We show that these molecules exhibit many of the characteristics of vertebrate collagens, including trimer formation and the presence of a collagen triple helical domain. They also contain a C-terminal trimerization domain, and a trimeric α-helical coiled-coil domain with an unusual amino acid sequence almost completely lacking leucine, valine or isoleucine residues. Intriguingly, these molecules show high thermal stability, with the collagen domain being more stable than those of vertebrate fibrillar collagens, which are much longer and post-translationally modified. Under the electron microscope, collagen-like proteins from *E. coli* O157:H7 show a dumbbell shape, with two globular domains joined by a hinged stalk. This morphology is consistent with their likely role as trimeric phage side-tail proteins that participate in the attachment of phage particles to *E. coli* target cells, either directly or through assembly with other phage tail proteins. Thus, collagen-like proteins in enterohaemorrhagic *E. coli* genomes may have a direct role in the dissemination of virulence-related genes through infection of harmless strains by induced bacteriophages.

## Introduction

Enterohaemorrhagic *E. coli* (EHEC) is responsible for gastrointestinal disorders in humans that range from abdominal pain and diarrhoea to haemorrhagic colitis and haemolytic uremic syndrome [1], [2], [3]. The EHEC serotype most often linked with outbreaks of severe disease is *E. coli* O157:H7. The genomes of the *E. coli* O157:H7 strains EDL933 and Sakai are 0.9 Mb larger than that of the non-pathogenic laboratory *E. coli* strain K-12 [4], [5]. That extra genetic material is the result of horizontal gene transfer (HGT) probably mediated by bacteriophages: the Sakai strain genome includes 18 prophages and 6 prophage-like elements integrated into different sites of the bacterial chromosome [5], [6], while the EDL933 genome contains 18 prophages and prophage-like elements [4]. Up to 463 phage-associated genes are present in the O157:H7 strains for only 29 in the K-12 strain [3], [7].

Several virulence genes of the O157:H7 strain are located into these prophages and prophage-like elements, notably the Shiga toxin (verocytotoxin) genes *stx1* and *stx2* [8], and various effector proteins that are injected into the host cells by a type III secretion system [9], [10]. Collectively, EHEC strains are considered new pathogens that have emerged from less virulent strains by progressive acquisition of virulence factors via HGT. There is significant evidence that variation of the prophage sequences is a main factor for the genomic and virulence diversity of EHEC [6], [7], [11], [12], [13]. The acquired specific virulent attributes allow EHEC strains to adapt to new niches and to broaden the spectrum of disease.

Intriguingly, the genomes of *E. coli* O157:H7 also include several open reading frames containing stretches of collagen-like sequences. Collagen proteins are principal components of the extracellular matrix of metazoa and amongst Earth’s most abundant biopolymers. Vertebrates have at least 28 collagen types described [14], with type I collagen being the main fibrous protein component of skin, tendon, bone and other connective tissues. All collagen proteins have at least one domain with a specific three-dimensional structure known as the collagen triple helix, in which three polypeptide chains wrap around a common helical axis and are connected through a ladder of intermolecular hydrogen bonds roughly perpendicular to that axis [15], [16], [17], [18]. The conformation of the collagen triple helix imposes a repetitive amino acid sequence pattern where glycine residues (Gly, G) occur at every third position. This (Gly-X-Y)*_n_* pattern is recognized as the signature of collagen proteins and domains.

A surprising number of collagen-like sequences have been detected outside the metazoan realm, notably in bacterial and viral genomes [19], [20], [21]. These “prokaryotic collagens” exhibit in their (Gly-X-Y)*_n_* regions significant differences in residue content and distribution with respect to vertebrate collagens, and yet they seem to show the basic molecular characteristics of true collagen proteins [22], [23]. The functions and potential contribution to virulence of these prokaryotic collagens are currently under study, but they seem to participate in pathogenesis in unexpected ways. Thus, collagen-like glycoproteins from *Bacillus anthracis* are components of the exosporium that are able to interact with integrin receptors on professional phagocytes [24], [25], while collagen-like surface proteins from *Streptococcus pyogenes* are able to promote bacterial adhesion and internalization to respiratory epithelial cells [26], [27], [28].

Open reading frames with collagen-like sequences in the genomes of *E. coli* O157:H7 and other EHEC strains are automatically annotated as “hypothetical tail fibre proteins”. These collagen-like sequences seem a distinctive feature of EHEC strains and several bacteriophages, and have not been detected in K-12 or other non-pathogenic strains. They are normally included in the prophage or prophage-like elements of the EHEC genomes and would be expected to participate in phage morphogenesis during prophage induction. Indeed there is evidence of changes in levels of expression for some of these collagen-like protein transcripts under certain experimental conditions, normally in association with other prophage genes [29], [30], [31], [32].

While most of the prophages in the EHEC genomes appear to be defective, often lacking genes apparently critical for phage induction and viability, phage induction from EHEC strains has been demonstrated and Shiga-toxin converting phages can be detected free in the extraintestinal environment [12], [33]. Furthermore, potentially defective phages have been shown to be inducible, to release virus particles of different morphologies and, after release, to infect other *E. coli* strains, [34]. The same study also suggests that recombination and other inter-prophage interactions may make possible the biological activation of defective prophages [34].

Thus, prophages embedded in EHEC genomes have the potential of disseminating virulence factors through bacterial infection and HGT. Their morphogenetic proteins are largely uncharacterized and deserve investigation. Here, we present a first biochemical analysis of the collagen-like proteins of EHEC prophages, which we will refer collectively as EPclPs (EHEC Prophage collagen-like Proteins).

## Results

### Domain Architecture of Collagen-like Proteins in EHEC Genomes

**Figure 1:**
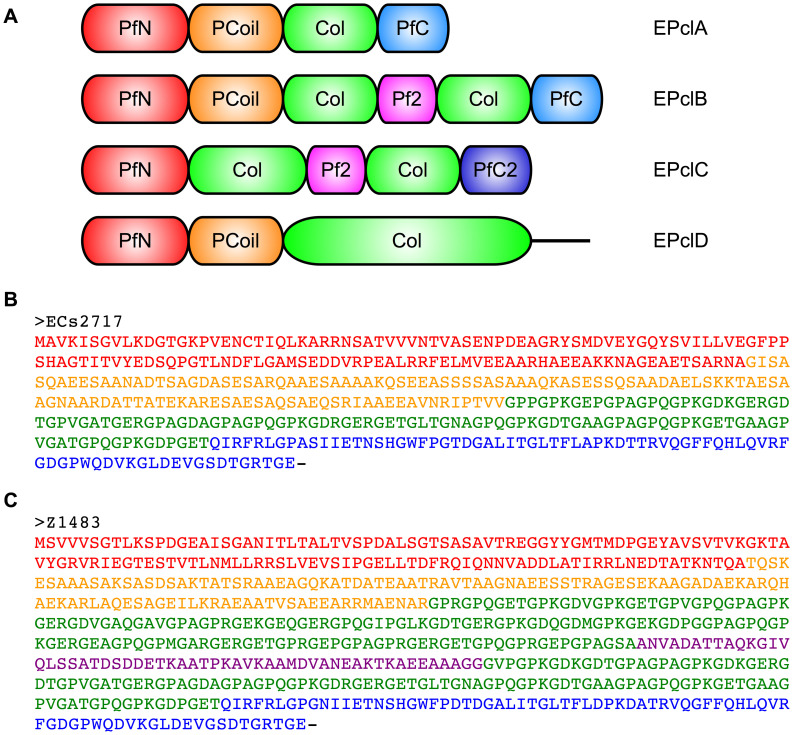
Collagen-like proteins from prophages embedded in the genomes of *E. coli* O157:H7 and other EHEC strains, referred here as EPclA to EPclD (EHEC Prophage collagen-like A to D). (**A**) Domain architectures. The collagen triple helical domains are labelled “Col”, and domains predicted to adopt an α-helical coiled-coil conformation (see text) are labelled “PCoil” (for phage coiled-coils). Key to other domain labels (Table 1): PfN, phage fibre N-terminal domain; PfC, phage fibre C-terminal domain; PfC2, phage fibre C-terminal domain, variant 2; Pf2, phage fibre repeat 2. (**B**) Sequence of a representative collagen-like protein with EPclA architecture (ECs2717), from the genome of *E. coli* O157:H7 Sakai. (**C**) Sequence of a representative collagen-like protein with EPclB architecture (Z1483), from the genome of *E. coli* O157:H7 EDL933. Amino acid sequences corresponding to the different predicted domains are colour-coded as in (**A**).

Several open reading frames potentially encoding collagen-like proteins have been identified by automatic sequence annotation in the genomes of EHEC strains. Those from the Sakai and EDL933 genomes will be discussed here, but many related sequences have been identified in other strains. Their primary structures show one or more collagen-like domains (Col) with the repeating collagen signature sequence (Gly-X-Y)*_n_*, flanked at both ends by a series of non-collagenous, conserved domains (Figure 1 and Table 1). Domains PfN, Pf2 and PfC have been described on the basis of sequence conservation and are associated to fibre tail proteins from phages. They appear in automatic annotation of EPclPs. Figure 1 shows the different protein architectures and the nomenclature used here to refer to them, plus two representative sequences. The most common architecture (EPclA) appears in multiple copies in each genome, with more than 90% amino acid sequence identity across copies. Table 2 gives the complete list of EPclP sequences from the Sakai and EDL933 genomes, whereas representative examples of other architectures and strains are given in Table 3.

**Table 1:** Domains observed in collagen-like proteins from the genomes of EHEC strains.

Domain	Names	InterPro	Pfam
Col	Collagen triple helix	IPR008160	PF01391
PCoil	Putative coiled-coil region (see text)		
PfN	Phage fibre N (Phage_tail_N; Prophage tail fiber N-terminal)	IPR013609	PF08400
PfC	Phage fibre C (Phage_fiber_C; Putative prophage tail fiber C-terminus)	IPR009640	PF06820
PfC2	Phage fibre C, variant 2 (see text)		
Pf2	Phage fibre 2 (Phage_fiber_2; Phage tail fiber repeat 2)	IPR005068	PF03406

Currently available accession codes on the InterPro and Pfam databases are given. Domain architectures are shown in Figure 1.

**Table 2:** Collagen-like proteins from the genomes of *E. coli* O157:H7 EDL933 and Sakai strains, and their corresponding prophage locations.

Strain	Gene name/locus	Type	Prophage	Length (aa)	PCoillength (aa)	Collagenlengths (aa)	Reference
O157:H7 EDL933	Z0982	EPclA	CP-933K	437	116	111	[4]
	Z1382	EPclA*	CP-933M	437	116	111	
	Z1483	EPclB	BP-933W	645	109	157, 114	
	Z2147	EPclA	CP-933O	437	116	111	
	Z2340	EPclA	CP-933R	437	116	111	
	Z3074	EPclA	CP-933U	437	116	111	
	Z3309, Z3307	EPclA*	CP-933V	437	116	111	
	Z6027	EPclA	CP-933P	437	116	111	
O157:H7 Sakai	ECs0844	EPclA	Sp3	437	116	111	[5]
	ECs1123	EPclA	Sp4	437	116	111	
	ECs1228	EPclB	Sp5	645	109	157, 114	
	ECs1808	EPclA	Sp9	437	116	111	
	ECs1992	EPclA	Sp10	437	116	111	
	ECs2159, ECs2158	EPclA*	Sp11	407	116	81	
	ECs2231	EPclA	Sp12	407	116	81	
	ECs2717	EPclA	Sp14	437	116	111	
	ECs2941	EPclA	Sp15	437	116	111	

* Defective protein sequence, with frame-loss between domains. The original gene sequence may correspond to two open reading frames.

**Table 3:** Examples of collagen-like proteins from other *E. coli* and *Shigella* strains, and their prophage locations.

Strain	Gene name/locus	Type	Phage/Prophage	Length (aa)	PCoillength (aa)	Collagenlengths (aa)	Reference
O157:H7 EC970520	YYZ_gp70	EPclA	YYZ-2008	389	116	63	[80]
O157:H7 EC508	ECH7EC508_5575	EPclA	U	407	116	81	[81]
	ECH7EC508_3568	EPclA	U	470	116	144	
O26:H11	ECO26_0887	EPclA	P02	404	116	78	[10]
	ECO26_1634	EPclA	P06	440	116	114	
O157:H7 Okayama	Stx2Ip020	EPclB	Stx2φ-1	678	109	190, 114	[82]
ECOR-9	ORF-401	EPclC	P-Eiba	479		117, 75	[83]
O81 ED1a	ECED1_1681	EPclC	U	493		106, 102	[84]
	ECED1_2081	EPclC	U	520		118, 117	
	ECED1_1138	EPclC*	U	566	95	48, 135	
55989/EAEC	EC55989_1078	EPclC*	U	467	116	140	[84]
O86:H–	Stx2-86_gp21	EPclD	Stx2-86	673	88	322	[85]
EC4100B	ECoL_05072	EPclD	U	519	116	135	[86]
*Shigella dysenteriae*	SDB_03138	EPclD	U	519	116	135	[87]
*Shigella boydii* BS512	SbBS512_E1096	EPclD	U	411	116	27	[88]

U: unassigned prophage.

* EPclC variant, containing a PCoil domain and either one or two collagen domains.

The EPclA architecture shows a single collagen triple helical sequence capped by PfN and PfC domains at the N- and C-termini, respectively. Between the PfN domain and the Col domain there is a region of low-complexity. Analysis of its amino acid sequence suggests a coiled-coil conformation (see below), and thus will be referred here as PCoil domain. The EPclB architecture shares the presence of PfN, PCoil and PfC non-collagenous domains, and contains two Col domains separated by a Pf2-type repeat. Protein sequences within each type of architecture show variable lengths of their Col and PCoil domains (Tables 2 and 3). Differences in length are typically multiples of three for Col domains and multiples of seven for PCoil domains, which is consistent with the lengths of the repetitive motifs in collagen and coiled-coil sequences, respectively. In sequences with two Col domains it is common that the first one contains a single interruption of the (Gly-X-Y)*_n_* repeating pattern, with a conserved Gly-X-Pro-Gly-Gly-Pro-X-Gly sequence.

Only a few sequences conform to the EPclC and EPclD architectures (Table 3), which are characterized by different C-terminal regions with no sequence homology to the PfC domains of EPclA or EPclB architectures. Also, the PCoil domain is often missing in EPclC architectures. The EPclD sequence Stx2-86_gp21, from the Stx2-86 prophage in the Shiga toxin-producing *E. coli* strain O86:H– (accession codes Q08J84, YP_794068), has a 322-amino acid Col domain (Table 3), by far the longest collagen-like sequence of all EPclPs. This long domain also shows a single Gly-X-Pro-Gly-Gly-Pro-X-Gly interruption. No examples of EPclC or EPclD sequences are found in the EDL933 or Sakai genomes.

The PfN and Pf2 domains are not exclusive to collagen-like proteins from *E. coli* prophages and were first identified in the side tail fibre protein coded by the *stf* gene from λ bacteriophages [35], [36] (accession code P03764). Virions with a functional *stf* gene show jointed tail fibres, expanded receptor specificity, and adsorb more rapidly to *E. coli* cells. Homologous proteins have been identified in embedded prophages of many *E. coli* strains, including the laboratory reference strain K12 (protein stfR/ynaB, accession code P76072).

Other than their consistent presence in prophage tail fibre proteins, little is known about the structure and function of the PfN and Pf2 domains. Some sequence similarity between PfN and a regulatory domain of eukaryotic carboxypeptidases may be indicative of a proteolysis-related function, but to date there is no experimental evidence for this. Due to the presence of these domains, EPclP sequences are automatically annotated as putative tail fibre proteins.

### Amino Acid Composition and Positional Preference in the Collagen Domains of EPclPs

Collagen domains in EPclPs show amino acid preferences in the X and Y positions that differ from those seen in other collagen proteins. By far the most common residue in the X position is proline (Pro, P), which occurs there close to half of the time (Table 4). By contrast, Pro is relatively infrequent in the Y position. Both X and Y positions also show a strong preference for charged amino acids, aspartate/glutamate (Asp/Glu, D/E) in the X position and lysine/arginine (Lys/Arg, K/R) in the Y position. Alanine (Ala, A) is also relatively frequent at both X and Y positions, and both glutamine (Gln, Q) and threonine (Thr, T) show a clear preference for the Y position. Interestingly, cysteine, phenylalanine, histidine, tryptophan and tyrosine are absent in collagen domains from EPclPs. At the triplet level, the most common are GPK (15%), GPQ (12%), GPA (12%), GER (8%) and GET (8%). The triplet pattern GP(Q/P)–GPK–G(D/E) is repeatedly observed in the collagen domains of EPclPs.

**Table 4:** Position-specific amino acid preferences in collagen triple-helical domains of EPclPs, human collagens, and collagen-like proteins from different groups of organisms.

Collagen group	X							Y						
	Pro	Asp,Glu	Arg,Lys	Ala	Gln	Thr	Other	Pro(Hyp)	Asp,Glu	Arg,Lys	Ala	Gln	Thr	Other
*EPclPs*	48.1	35.6	0.3	10.3	0.1	0.1	5.5	7.4	0.1	31.0	21.2	14.5	20.2	5.7
*Viruses* *	22.1	35.3	6.0	7.1	1.5	3.1	24.9	9.3	7.8	38.1	5.5	13.0	8.9	17.4
*Bacteria, gram positive*	31.0	14.1	5.1	23.1	2.6	1.4	22.7	3.7	5.9	9.7	6.2	18.4	48.3	7.8
*Human collagens*														
Fibrillar	31.1	18.5	6.6	9.8	3.4	1.6	28.9	33.5	6.9	21.3	9.1	7.3	4.1	17.8
Non-fibrillar	24.6	19.7	6.6	5.8	4.1	2.4	36.9	42.2	5.4	22.4	4.7	6.5	2.8	16.0
*Human collagen-like proteins* †	27.5	21.6	10.6	6.1	3.0	2.0	29.2	33.7	4.8	26.1	5.3	7.9	3.5	18.8

The X and Y letters refer to the consensus sequence pattern (Gly-X-Y)*_n_* characteristic of collagen triple-helical domains. The numbers indicate percentage occupation of the X or Y position by a given amino acid type.

* Excluding EPclPs from bacteriophages.

† Include molecules such as C1q, mannose binding proteins, collectins, macrophage scavenger receptors, or acetyl cholinesterase, which contain in their sequence a collagen domain but are not formally classified as collagen types.

The position-specific amino acid preferences in EPclPs are quite different from those seen in animal collagens, as shown for example by the human sequences (Table 4 and [37]). The most obvious difference is in the Pro distribution: human collagens have a clear preference for Pro residues in both the X and Y positions, close to 30% and 35% respectively. There is some variation between fibrillar and non-fibrillar collagens and with the collagen-like proteins, but Pro residues are invariable more common in the Y position of human collagens. The reason is well known: Pro residues in the Y position are often modified post-translationally to 4-hydroxyproline (Hyp, O), which contributes to the thermal stability of the collagen domains. Charged residues are also frequent in the X and Y positions of human collagens, with the same positional preferences as in collagen domains from EPclPs (Asp/Glu more often in X, Lys/Arg more often in Y). However, they are overall less frequent and their preferential position is less strict (Table 4). Other amino acids significantly contribute to the sequence variability at each position.

The expected average conformational parameters of the triple helical Col domains can be calculated from the distribution of imino acids along their sequences [38]. The expected values are –106° for the average twist and 2.88 Å for the average height, same as those predicted for human fibrillar collagens [38]. Thus, despite the differences in amino acid composition and positional preference, the overall conformation of the triple helical Col domains is expected to be very similar to that of human fibrillar collagens.

Collagen sequences found in other viral proteins are more similar to those from EPclPs, although the preference for Pro in the X position is not that strong. Viral collagens share with EPclPs the low proportion of Pro residues in the Y position, large number of charged amino acids, and relatively common occurrence of Gln and Thr in the Y position. Collagens from gram-positive bacteria, which include the well-studied examples of *Bacillus anthracis* or *Streptococcus pyogenes* [22], [24], [39], show a lower presence of Pro residues in the X and Y positions, much lower proportion of charged amino acids, and a higher proportion of Ala residues in the X position and Gln and Thr in the Y position.

The main difference between human collagens and the three groups of non-animal collagens in Table 4 is the lack of preference for Pro in the Y position (as already noted in an earlier analysis of viral and bacterial collagen structural motifs [21]). Bacteria and viruses do not have the prolyl-hydroxylase enzymes required for hydroxylation of Pro in the Y position of a collagen triple helix, and therefore there is no contribution to the stability of their collagen domains by Hyp residues. Collagen domains from EPclPs appear to compensate this lack of prolyl hydroxylation with a larger proportion of Pro residues in the X position. The high ratio of charged amino acids and the relatively high occurrence of Ala, Gln and Thr in EPclPs and bacterial and viral collagens may be indicative of different mechanisms for stability of their collagen domains [40], [41], [42].

Interestingly, the metazoan collagen sequence closest to EPclPs comes from a sea anemone, *Nematostella vectensis* (NCBI accession code XP_001625905, incomplete sequence), with 56% identity to the collagen domain of the EPclA protein ECs2717 and containing a repetitive [GP(Q/E)-GPK-GDT-GIT]_12_ sequence, reminiscent of the commonly observed triplet pattern mentioned above.

### A Low Complexity Region is Predicted as α-helical Coiled-coil Domain (PCoil)

**Figure 2:**
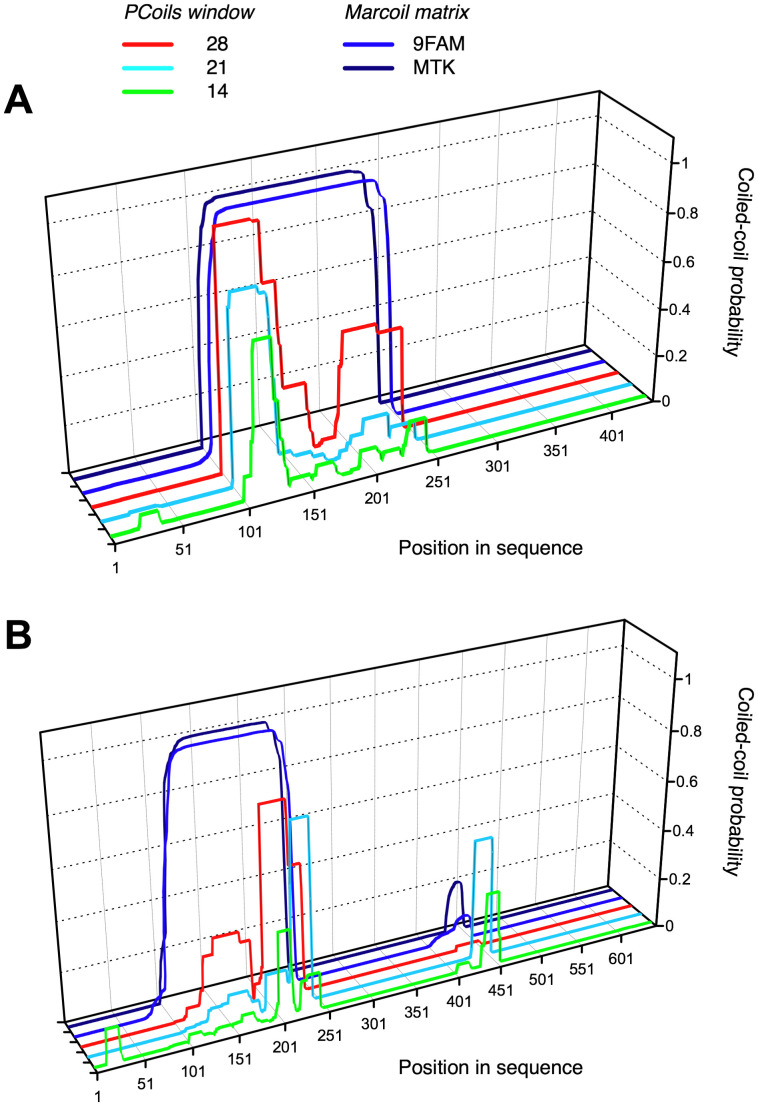
Coiled-coiled predictions for the amino acid sequences of (A) EPclA (ECs2717) and (B) EPclB (ECs1228), using the *PCoils* [70] and *Marcoil* [71] algorithms. The graphs indicate regions of high probability for α-helical coiled-coil formation. Three different sequence window sizes were used with the *PCoils* algorithm: 14, 21 and 28 residues. Two different matrices were used in *Marcoil*: 9FAM, and MTK-based.

The region between the predicted PfN and Col domains in the most common architectures, EPclA and EPclB, shows an unusual low-complexity sequence with predominance of Ala (32%), Ser (19%) and Glu (13%) amino acids that often appear in tandems or in stretches of up to four consecutive identical residues (Figure 1). Different coiled-coil predicting algorithms (*PCoils*, *Marcoil*, *MultiCoil*) give high scores for the region between residues 101 and 245 in both EPclA and EPclB (Figure 2). This region includes the low-complexity sequence between the PfN and Col domains plus the last 34 residues of PfN, and shows a loose seven-residue Ala-X-X-Ala/Ser-X-X-Ser periodicity, where residues in the X positions are often charged. On account of the coiled-coil predictions we will refer to the low-complexity region between PfN and Col as the PCoil domain. The *MultiCoil* and *SCORER 2.0* prediction algorithms favour a trimeric rather than dimeric coiled-coil structure for PCoil. Secondary structure prediction by *Jpred3* suggests that the PfN domain has mainly a β-sheet structure for the first 80 residues and some α-helical conformation from residues 90 onwards, whereas the PCoil region is predicted to be mainly α-helical. *Jpred3* does not predict any secondary structure for the PfC domains (data not shown).

### EPclA is a Trimeric Protein that Dissociates When Denatured

**Figure 3:**
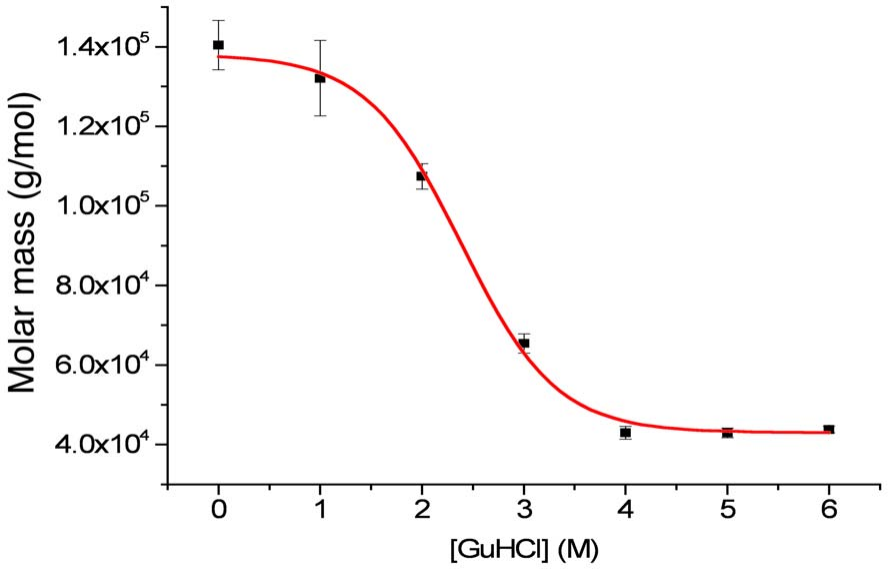
Analysis by analytical ultracentrifugation of the average molar mass of a sample of purified *r*EPclA as a function of increasing concentration of guanidinium chloride (GuHCl). Weight-averaged molar mass was determined using a single ideal species model (see Methods). Mean value masses for the upper and lower plateaux were 138±6 kDa and 43±1 kDa respectively (averages of three measures). The molecular mass of native *r*EPclA (0 M GuHCl) is consistent with three times that of denatured *r*EPclA (see text). The transition midpoint concentration is 2.38±0.09 M GuHCl.

The quaternary structure of *r*EPclA was investigated by sedimentation equilibrium analytical ultracentrifugation (AUC) at increasing concentrations of guanidinium chloride (GuHCl) (Figure 3). The relative molar mass of *r*EPclA at 0 M GuHCl was 138±6 kDa, corresponding to the predicted molecular weight of a trimer of *r*EPclA molecules (3×47 kDa). As the concentration of GuHCl increased, a transition from trimer to monomer was observed and the relative molar mass of *r*EPclA at 5 M GuHCl was 43 kDa, which is consistent with the predicted molecular weight of the *r*EPclA monomer. Thus, *r*EPclA trimers dissociate into monomers as the GuHCl concentration increases; the trimer-to-monomer transition point was estimated at around 2.5 M GuHCl.

An independent measurement of the molecular weight of *r*EPclA was carried out by size exclusion chromatography followed by multiangle laser light scattering (SEC/MALLS) (Figure S1). The molecular weight obtained from MALLS is consistent with a trimer of *r*EPclA (Table 5). A proteolytic fragment from *r*EPclA that included only the Col and PfC domains (Col–PfC fragment, Figures S2 and S4) could be produced in enough amounts for biophysical characterization. Analysis by SEC/MALLS of fractions containing the Col–PfC fragment (Figure S1) showed it to be trimeric as well (Table 5), with a molecular weight of 64 kDa consistent with three times the molecular weight of monomer Col–PfC (21–22 kDa, predicted from the peptide fingerprinting data obtained from mass spectrometry, Figure S2).

**Table 5:** Average molecular weights of different recombinant fragments, calculated from the SEC/MALLS data.

Molecule	Average *Mw* (kDa)SEC/MALLS	Predicted *Mw* (kDa)from sequence	Oligomer state
*r*EPclA	145	47.3	Trimer
Col–PfC	62	∼21	Trimer
PfN–PCoil	88	28.0	Trimer
PfN	17	17.2	Monomer
Trx–PfC	64	21.0	Trimer
PfC	24	7.2	Trimer

Domain compositions of each molecule, including protein fusion tags, are shown in Supplementary Figure S5.

The molecular weights obtained from AUC and MALLS experiments were consistent with the predicted values for non-glycosylated *r*EPclA trimers and monomers. Lack of glycosylation of *r*EPclA was confirmed by periodic acid-Schiff staining analysis (data not shown).

### Domains PCoil and PfC from EPclA are Trimerization Modules

Molecular weights of several recombinant fragments containing different combinations of domains were determined by SEC/MALLS (Figure S3 and Table 5). The data indicates that PfC is a trimerization domain, forming trimeric assemblies both when fused to a thioredoxin tag (Trx–PfC) or after removal of thioredoxin by thrombin digestion. The PfN–PCoil fragments were also trimeric, whereas the PfN domains were mainly in the monomer state (Figure S3). This data suggests that PCoil is also a trimerization domain.

### EPclA Shows a CD Spectrum Consistent with Collagen and α-helical Conformations

**Figure 4:**
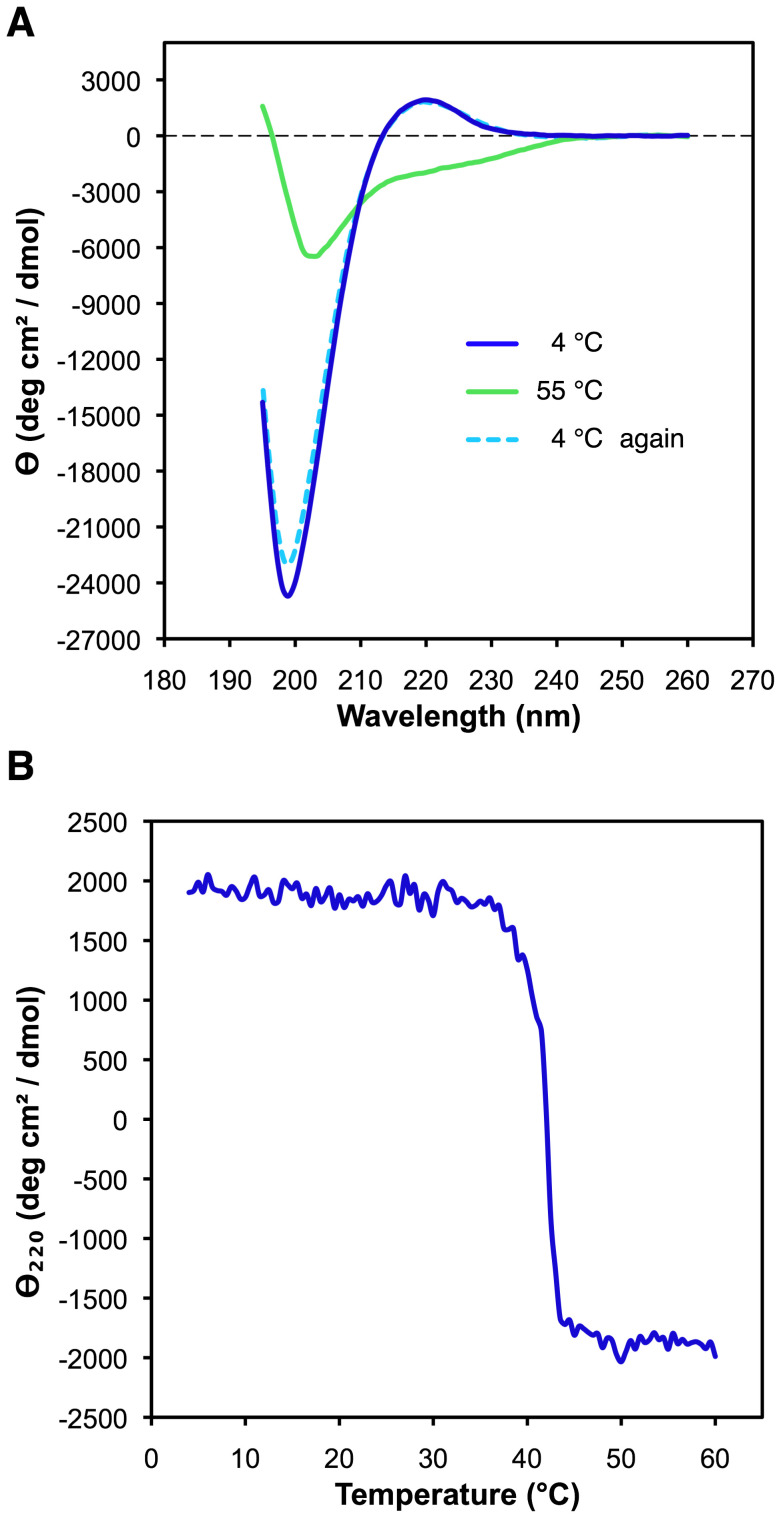
Far-UV CD analysis of the Col–PfC fragment after purification from *r*EPclA by SEC. (**A**) CD spectra at 4°C, 55°C and 4°C after immediately cooling back the sample (see text). The vertical axis measures mean residue ellipticity Θ in degrees cm^2^ dmol^-1^. The CD data was collected between 195 and 260 nm, with a protein concentration of 0.2 mg/ml in 10 mM Tris, 150 mM NaCl, pH 7.4. Measurements were taken in a 0.5 mm path length cell. (**B**) Thermal denaturation of the Col–PfC fragment, monitored by CD at 220 nm as a function of increasing temperature between 4°C and 60°C, with a protein concentration of 0.2 mg/ml in 10 mM Tris, 150 mM NaCl, pH 7.4, and a heating rate of 0.33°C/min.

The secondary structures of *r*EPclA and the Col–PfC fragment were analysed by circular dichroism (CD). Interpretation of the results is easier if the Col–PfC CD data is considered first. A sample of Col–PfC was purified from a preparation of full-length *r*EPclA by nickel-affinity and size exclusion chromatographies. Its CD spectrum was measured at different temperatures between 195 and 260 nm. The concentration of the Col–PfC sample was calculated as 0.2 mg/ml from its UV absorption at 280 nm and an estimated molar extinction coefficient ε = 11000 M^-1^ cm^-1^. The CD spectrum of Col–PfC at 4°C (Figure 4A) shows the characteristic features of triple helical collagen: a small maximum of positive ellipticity at 220 nm and a deep minimum of negative ellipticity at around 199 nm [43]. Both these features are associated with the polyproline II conformation [44] characteristic of the collagen triple helix. The Col–PfC fragment includes mainly the collagen domain (Col) of EPclA and the C-terminal PfC domain, and thus its CD spectrum suggests that the Col domain adopts indeed a collagen-like, triple helical structure. The triple helical features disappeared from the CD spectrum upon increase of temperature, as shown by the CD curve at 55°C (Figure 4A). Interestingly, immediate cooling of the same sample back to 4°C recovered completely the triple helical structure, with a CD spectrum practically indistinguishable from the initial one (Figure 4A).

To examine the thermal denaturation of the collagen triple helix in the Col–PfC domain, the CD of another sample of purified Col–PfC fragment (also 0.2 mg/ml) was monitored at 220 nm as a function of continuously increasing temperature, from 4°C to 60°C. The thermal curve showed a single sharp transition at 42°C, which typically corresponds to the decrease of ellipticity at 220 nm and loss of collagen triple helical structure (Figure 4B).

**Figure 5:**
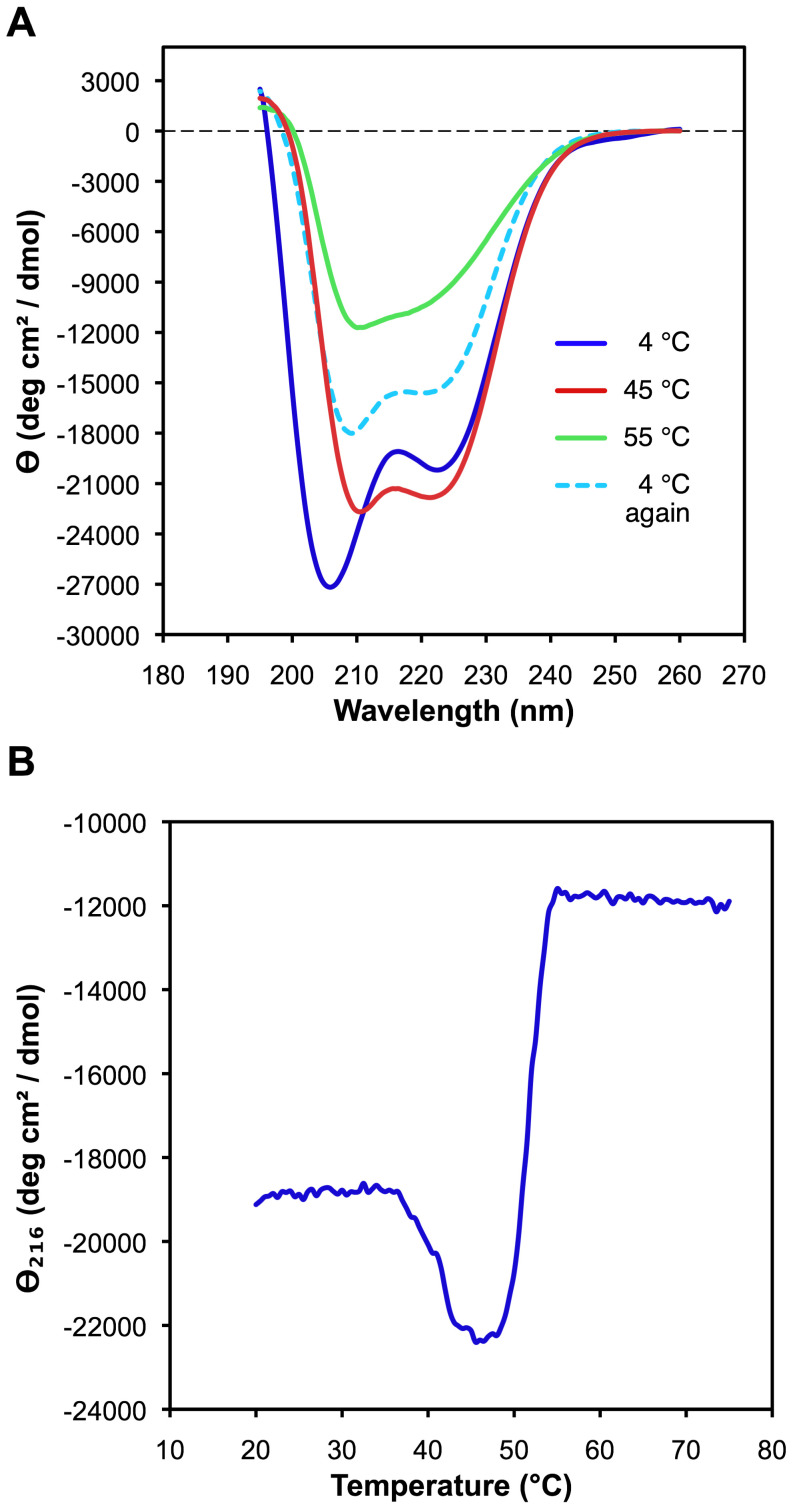
Far-UV CD analysis of *r*EPclA after purification by SEC. (**A**) CD spectra at 4°C, 45°C, 55°C and 4°C after immediately cooling back the sample (see text). The vertical axis measures mean residue ellipticity Θ in degrees cm^2^ dmol^-1^. The CD data was collected between 195 and 260 nm, with a protein concentration of 0.04 mg/ml (4°C) or 0.3 mg/ml (the rest) in 10 mM Tris, 150 mM NaCl, pH 7.4. Measurements were taken in a 0.5 mm path length cell. (**B**) Thermal denaturation of *r*EPclA monitored by CD at 216 nm (the maximum between the two minima at 208 and 224 nm). The CD was measured as a function of increasing temperature between 20°C and 75°C, with a protein concentration of 0.3 mg/ml in 10 mM Tris, 150 mM NaCl, pH 7.4, and a heating rate of 0.33°C/min.

The CD spectrum of *r*EPclA is different. A diluted sample of *r*EPclA was purified by nickel-affinity and size exclusion chromatographies and its CD spectrum was measured between 195 and 260 nm at 4°C (Figure 5A). The concentration of the *r*EPclA sample was calculated as 0.04 mg/ml from its UV absorption at 280 nm and a molar extinction coefficient ε = 17000 M^-1^ cm^-1^, deduced from the amino acid sequence of *r*EPclA. The CD spectrum showed two minima of negative ellipticity at 205 nm and 224 nm, the first one being deeper, and a small local maximum between the two minima, at 216 nm. To investigate this region in more detail a second sample with higher concentration, 0.3 mg/ml, was analyzed at different temperatures. When the sample was heated to 45°C, the height of the 216 nm maximum decreased significantly, the intensities of the two minima became more similar to each other, and their positions shifted to 210 nm and 222 nm, respectively (Figure 5A). This spectrum resembled more that of an α-helical coiled-coil conformation. Upon further increase of the temperature the overall ellipticity became less negative, and the two minima started to disappear and vanished completely when reaching 55°C (Figure 5A). The spectrum did not change upon further increase of temperature to 65°C. The slight decrease in ellipticity at 216 nm around 45°C and the more similar intensities of the two minima at that temperature suggest changes in the secondary structure that are consistent with the loss of the triple helical conformation in the Col domain while maintaining an α-helical conformation (Figures 4 and 5). Such α-helical structure appears to be more stable and does not disappear completely until 55°C. Immediate cooling of the same sample from 65°C back to 4°C recovered approximately half of the initial CD spectrum (Figure 5A).

To examine the thermal denaturation of *r*EPclA, the CD of another sample of purified *r*EPclA (concentration 0.3 mg/ml) was monitored at 216 nm as a function of increasing temperature from 20°C to 75°C. Two transitions were observed: a first transition at 42°C showed a sharp decrease in ellipticity, consistent with the loss of collagen triple-helical structure seen previously for the Col–PfC fragment at the same temperature; a second transition at 52°C showed a sharp and pronounced increase in ellipticity, corresponding to the loss of α-helical structure of the PCoil and PfN domains (Figure 5B). Thus, the α-helical structure of the PCoil and PfN domains is more stable than the collagen triple helix of the Col domain. The transition temperature of the Col domain is the same in *r*EPclA and its Col–PfC fragment, and seems unaffected by the presence of the PCoil and PfN domains. The melting transitions of the α-helical and collagen structures therefore appear to be largely independent.

### The PfN–PCoil Region is Clearly α-helical and is Consistent with a Coiled-coil Structure

**Figure 6:**
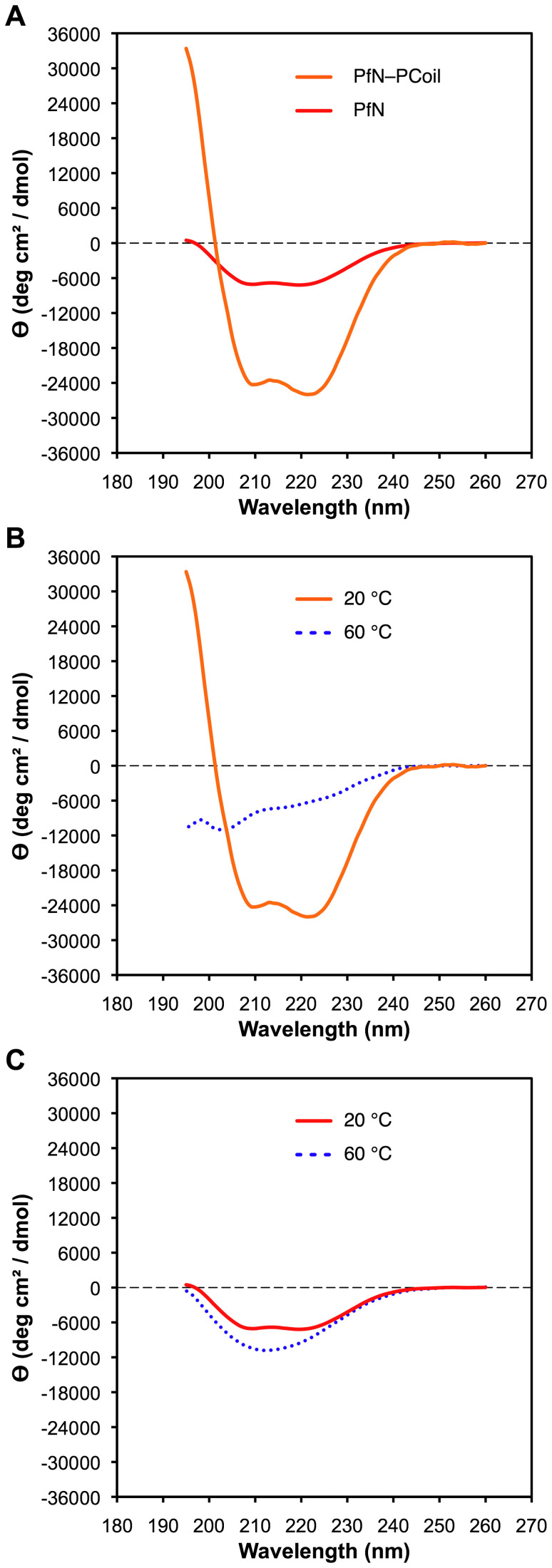
Far-UV CD spectra of the PfN–PCoil and PfN fragments after purification by SEC: (**A**) **PfN and PfN–PCoil at 20°C;** (**B**) **PfN–PCoil at 20°C and 60°C;** (**C**) **PfN at 20°C and 60°C.** In all panels the vertical axis measures mean residue ellipticity Θ in degrees cm^2^ dmol^-1^. The CD data was collected between 195 and 260 nm, with protein concentrations of 0.2 mg/ml (PfN–PCoil) or 0.3 mg/ml (PfN), in 20 mM phosphate buffer (Na_2_HPO_4/_NaH_2_PO_4_), 100 mM NaCl, pH 7.4. Measurements were taken in a 0.5 mm path length cell.

The secondary structures of the recombinant fragments PfN–PCoil and PfN were also studied by CD spectroscopy. Recombinant PfN–PCoil and PfN fragments were each purified with nickel-affinity and size-exclusion chromatographies. Concentrations of the PfN–PCoil and PfN samples were measured as 0.2 mg/ml and 0.3 mg/ml respectively, from their absorption at 280 nm and molar extinction coefficients ε = 7000 M^-1^ cm^-1^, initially calculated from the amino acid sequences of the PfN–PCoil and PfN recombinant fragments and adjusted using the observed UV absorption of samples in 8 M urea (see Materials and Methods). The CD spectrum of PfN–PCoil at 4°C in phosphate buffer (Figure 6A) shows the characteristic features of an α-helical protein, with two minima at 208 nm and 222 nm and a maximum at 195 nm. This spectrum is consistent with the prediction of an α-helical coiled-coil conformation for the PCoil region. The α-helical features were still present in a spectrum measured at 45°C, although the signal intensities at the two minima started to decrease (data not shown). These features disappeared when reaching 60°C (Figure 6B), and were mostly recovered upon cooling the sample back to 20°C (data not shown). The spectrum of the PfN domain (Figure 6A) was also consistent with an α-helical structure but the intensity of the two minima at 208 nm and 222 nm was much lower than in the PfN–PCoil spectrum, indicating a lower α-helical content in this domain. This spectrum also vanished at 60°C (Figure 6C), but it was not recovered upon cooling back to 20°C (data not shown). Thus, the main contribution to the CD signal comes from the PCoil domain, most likely through the formation of a trimeric α-helical coiled-coil structure that disappears at a temperature of 60°C but is regained when the temperature is lowered again.

**Figure 7:**
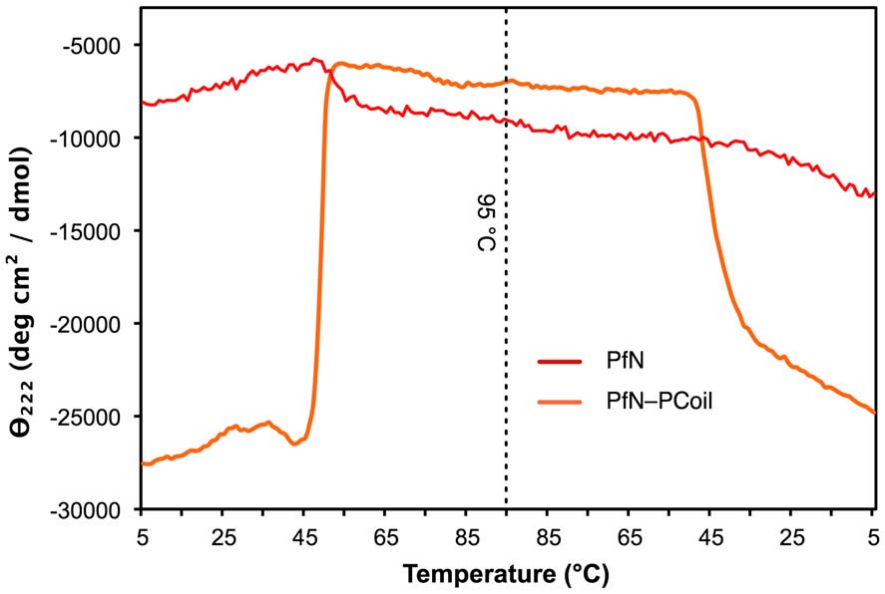
Thermal denaturation and renaturation of recombinant PfN–PCoil (orange) and PfN (red) monitored by CD at 222 nm (corresponding to a minimum in both CD spectra). The CD was measured in a 1 mm path length cell as a function of increasing temperature between 5°C and 95°C (left) and then decreasing temperature between 95°C and 5°C (right). The temperature was changed at a rate of 1°C per minute. Both PfN–PCoil (0.35 mg/ml) and PfN (0.1 mg/ml) were in 10 mM Tris, 150 mM NaCl, pH 7.4. PfN–PCoil showed a sharp transition at around 49°C corresponding to the loss of α-helical coiled-coil structure. The CD signal was almost completely recovered upon cooling, with a sharp transition about 45°C. This behaviour is indicative of a reversible structural transition for the α-helical coiled-coil. The PfN fragment gradually lost its CD signal with a transition midpoint of about ∼52°C. The gradual nature of this transition suggests denaturation rather than a cooperative unfolding. The PfN CD signal was not regained upon cooling.

To examine the thermal transitions of the PfN–PCoil domain, the CD of two more samples of purified recombinant PfN–PCoil (0.35 mg/ml concentration) and PfN (0.1 mg/ml) were monitored at 222 nm as a function of continuously increasing temperature, from 5°C to 95°C and then cooling back to 5°C at the same speed (1°C per minute). The heating thermal curve showed a single sharp transition at around 49°C (Figure 7), corresponding to the loss of the strong α-helical CD spectrum. The cooling thermal curve also showed a single sharp transition at around 45°C indicating partial re-gaining of the α-helical structure (the baseline in Figure 7 does not recover its initial value). This data suggests reversibility for the thermal transition of PfN–PCoil, mainly for the formation of the trimeric α-helical coiled-coil structure in the PCoil domain; the PfN domain does not recover completely after thermal denaturation and slow cooling. The actual value of the temperature of the transition is sensitive to the heating or cooling rate. A repeat of the heating experiment with a slower speed (0.33°C per minute) gave a transition temperature of 52°C for the PfN–PCoil fragment (data not shown).

### Structural Organization of rEPclA and its Fragments Col–PfC and PfN–PCoil

**Figure 8:**
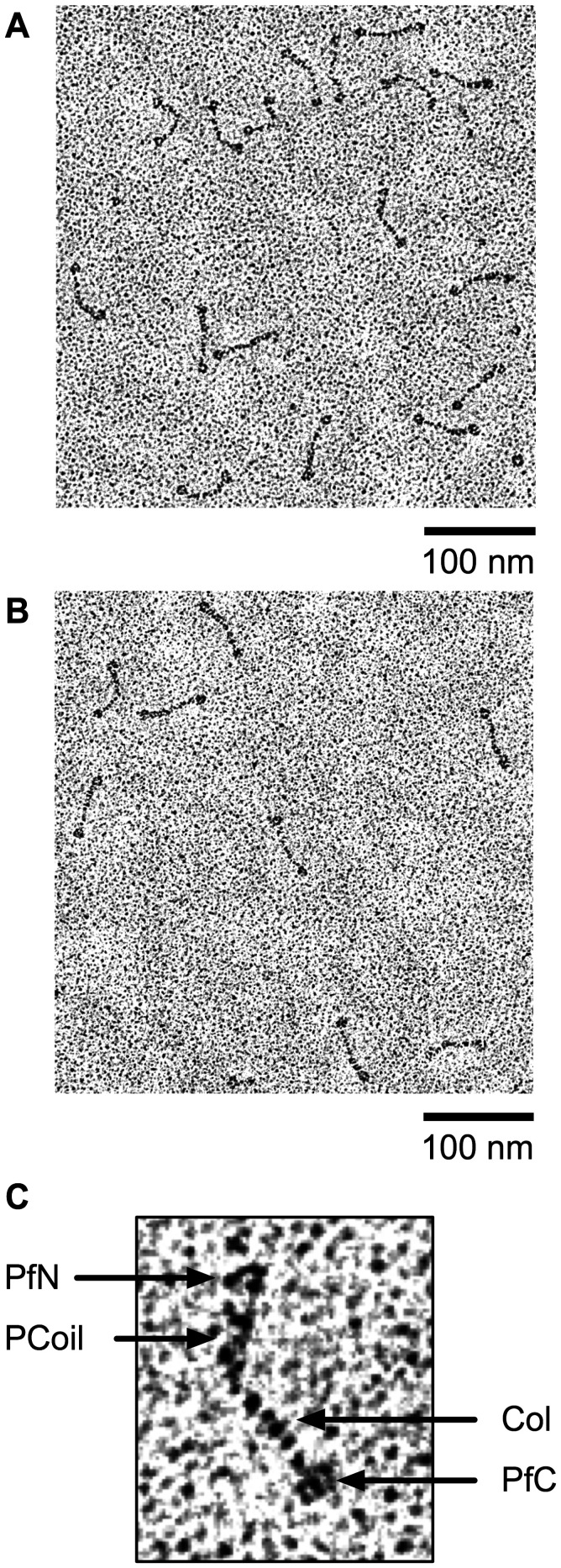
Rotary shadowing electron microscopy of *r*EPclA. (**A, B**) Different micrographs showing dumbbell-shaped structures corresponding to *r*EPclA trimers. The globular shapes correspond to the PfN and PfC terminal domains, presumably forming trimeric structures themselves. Flexible stalks connecting these globular structures contain the trimeric collagen triple-helical region (Col) and the trimeric α-helical coiled-coil region (PCoil) of *r*EPclA. The concentration of *r*EPclA was 5 µg/ml. Scale bar  = 100 nm. (**C**) Detailed view of an *r*EPclA trimer. The arrows indicate the globular terminal domains and the thin (Col) and thick (PCoil) regions of the stalk, respectively. The globular domains can be identified as N- or C-terminal by their position with respect to the two stalk zones.

**Figure 9:**
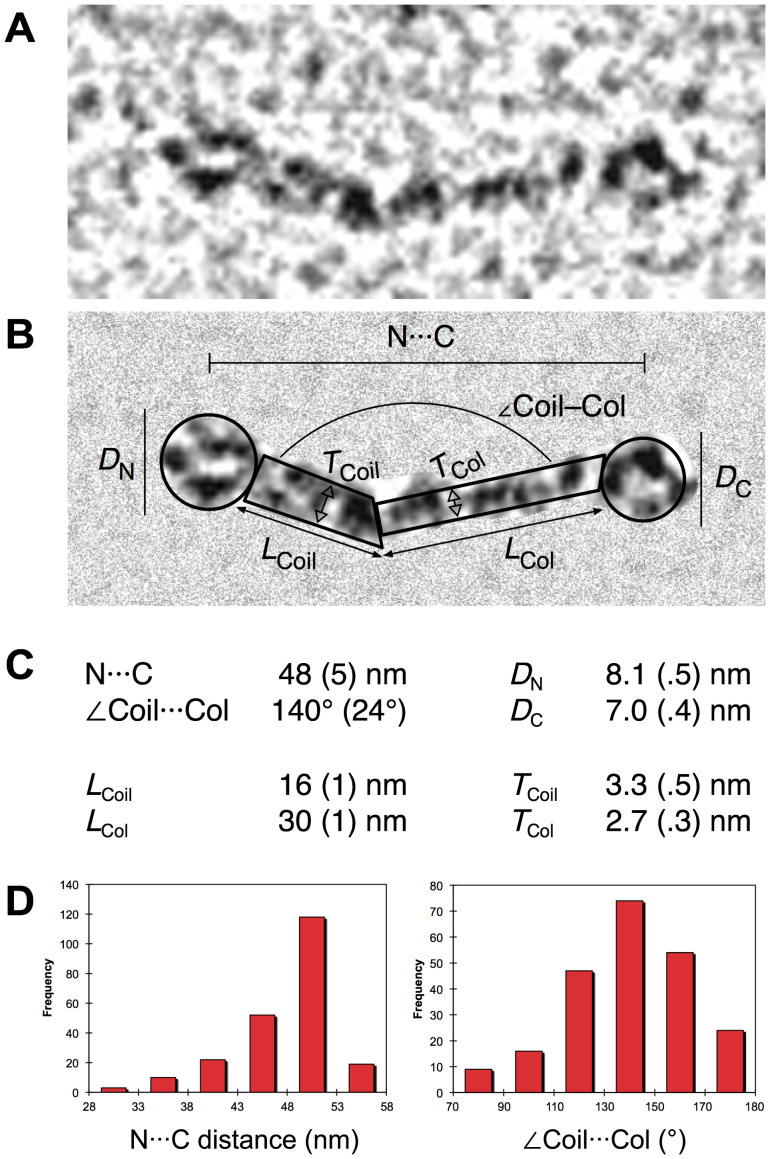
Molecular dimensions of *r*EPclA and its domains. (**A**) Magnified view of a representative *r*EPclA molecule from a rotary shadowing electron micrograph. (**B**) The same molecule with the background masked out showing the different molecular dimensions analyzed below. (**C**) Average dimensions obtained from multiple measures on electron micrographs: N···C and <Coil···Col are averages of 224 measures on eight *r*EPclA micrographs; *D*_C_, *L*_Col_ and *T*_Col_ are averages of 76, 35 and 74 measures, respectively, on three Col–PfC micrographs (Figure 10A); *D*_N_, *L*_Coil_ and *T*_Coil_ are averages of 200 measures on one PfN–PCoil micrograph (Figure 10B). (D) Histograms showing the distribution of N···C and <Coil···Col values on the sample of 224 *r*EPclA molecules.

Full-length *r*EPclA was analyzed by rotary shadowing electron microscopy. Examination of the electron micrographs of *r*EPclA showed a “dumbbell-shaped” structure with two globular particles joined by a semi-flexible stalk, in which it is possible to distinguish two regions of different thickness (Figures 8 and 9). Sequence analysis of the PCoil region and the CD spectrum of PfN–PCoil both suggest that the PCoil domain is a trimeric α-helical coiled-coil structure, and the observed thicker region of the stalk is consistent with such coiled-coil helical structure, which is known to have a larger cross-section than a collagen triple helix [45]. The remaining thinner region corresponds to the collagen triple-helical domain.

**Figure 10:**
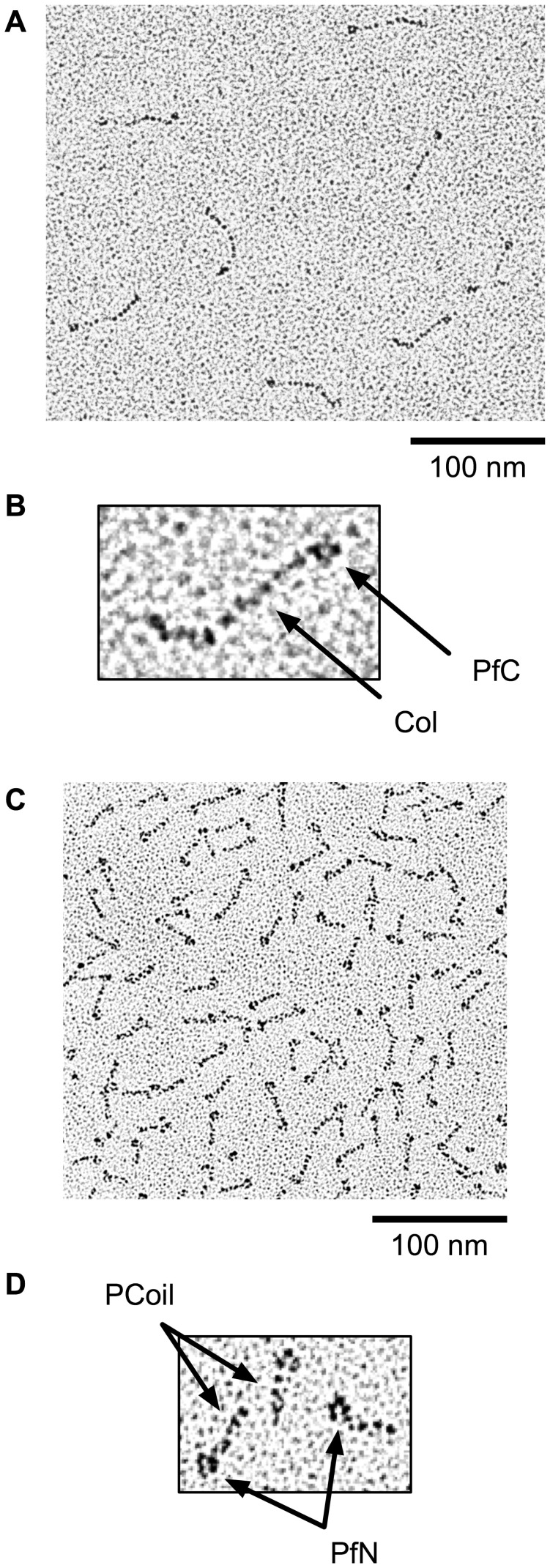
Rotary shadowing electron microscopy of Col–PfC and PfN–PCoil fragments. (**A**) Micrograph showing the morphology of Col–PfC fragments (concentration 1 µg/ml). (**B**) Detailed view of one Col–PfC fragment. The globular shape corresponds to a trimer of PfC domains and the stalk corresponds to the trimeric collagen triple-helical domain (Col). The N-terminal end of the stalk shows a short, unravelled tail, where the PfN and PCoil domains have been removed. (**C**) Micrograph showing the morphology of PfN–PCoil fragments (concentration 5 µg/ml). (**D**) Detailed view of three PfN–PCoil fragments. The globular shapes correspond to trimers of PfN domains and the short tails correspond to the PCoil domains.

Inspection of *r*EPclA molecules from different micrographs suggests a hinge between the two regions of the stalk (collagen and coiled coil) that results in variable angles between the PCoil and Col domains and variable distances between the two globular domains (Figure 9). For most *r*EPclA molecules the distance between the centres of the PfN and PfC domains (N···C in Figure 9) has values around 50 nm, reaching 55 nm for the most extended, linear ones. The average N···C distance (48±5 nm) is probably less significant than the values shown by the extended molecules. The N-terminal globular structure, made of three PfN domains, has an approximate diameter of 8 nm and is slightly bigger than the C-terminal globular structure made of three PfC domains (approximate diameter 7 nm) (Figure 9). Taking into account the radii of the globular domains, *r*EPclA molecules can reach a length of ∼62 nm when totally extended. However, most *r*EPclA molecules appear slightly bent at the hinge between the PCoil and Col domains and are, overall, a bit shorter (55–60 nm, including the globular domains).

Rotary shadow electron micrographs of Col–PfC fragments showed molecules very reminiscent of those of *r*EPclA but with only one globular domain (PfC) connected to a stalk (Col) (Figure 10A,B). The stalk region appears slightly unravelled where the α-helical coiled coil and the PfN domain have been removed by endogenous proteolysis of full-length *r*EPclA. The observed morphology confirms the previous assignment of N- and C-terminal domains for *r*EPclA images and that the thin region of the stalk corresponds to the collagen triple helix.

Rotary shadowing electron micrographs of PfN–PCoil fragments identified molecular shapes consistent with the N-terminal half of *r*EPclA molecules (Figure 10C,D). PfN domains (approximately the first 134 residues of EPclA) form a trimeric globular structure attached to an elongated stalk containing the PCoil domain. The micrographs show several instances of PfN–PCoil fragments apparently joined at the tails of their PCoil domains (Figure 10C). This association may result from some interaction between partially unravelled or unfolded chains at the terminal end of the PCoil domains.

From measures on the electron micrographs of the Col–PfC and PfN–PCoil fragments it is possible to estimate the lengths of the PCoil and Col regions as approximately 16 nm and 30 nm respectively (Figure 9). The length of the collagen domain is consistent with the predicted length of a collagen triple helix of 111 residues (111×2.9 Å = 32 nm, where 2.9 Å is an approximate measure of the height of an individual residue in a collagen triple helix [38]. Similar estimates can be obtained from measures on the thick and thin regions of the stalk in the *r*EPclA micrographs (14 nm for the PCoil region and 28 nm for the Col domain). The slightly shorter values suggest some overlap between domains in the *r*EPclA stalk that cannot be resolved in the rotary shadowing micrographs. The apparent thickness of the PCoil and Col domains in the electron micrographs over-estimates the true cross-section dimensions of these domains, as it includes the thickness of the shadowing replica. Nevertheless, a slight difference is observed between these estimates for the PCoil (3.3±0.5 nm) and Col (2.7±0.3 nm) domains. Cross-section values seen in high-resolution structures of these domains are closer to 1.3–1.7 nm for a collagen triple helix and 2.2–2.7 nm for a trimeric α-helical coiled coil.

### Structural Organization of rEPclB High-molecular Weight Aggregates

Expression of *r*EPclB both by IPTG induction or auto-induction produced a good yield of soluble protein. However, purification of *rEPclB* from the soluble fraction by nickel-affinity and size exclusion chromatography showed that all the protein went to form soluble, high-molecular weight aggregates that eluted in the void volume of the size exclusion columns (data not shown). The protein aggregated to such an extent that it was not possible to identify any additional peak or shoulder suitable for molecular mass determination by MALLS. Aggregation was worse in samples produced by auto-induction (presumably due to the increased protein production). Attempts to reduce the degree of aggregation by lowering the protein concentration, adding EDTA (to rule out His_6_-mediated metal chelation aggregation), adding glucose in high concentration, or changing the ionic strength of the buffers, were all unsuccessful: it was not possible to obtain enough non-aggregated *r*EPclB for molecular weight determination or for CD studies. Interestingly, the high molecular weight aggregates were soluble and the protein did not precipitate out of solution, even after high-speed centrifugation. The bands observed in SDS-PAGE experiments suggest that SDS treatment extracts monomeric *r*EPclB from the high molecular weight aggregates.

**Figure 11:**
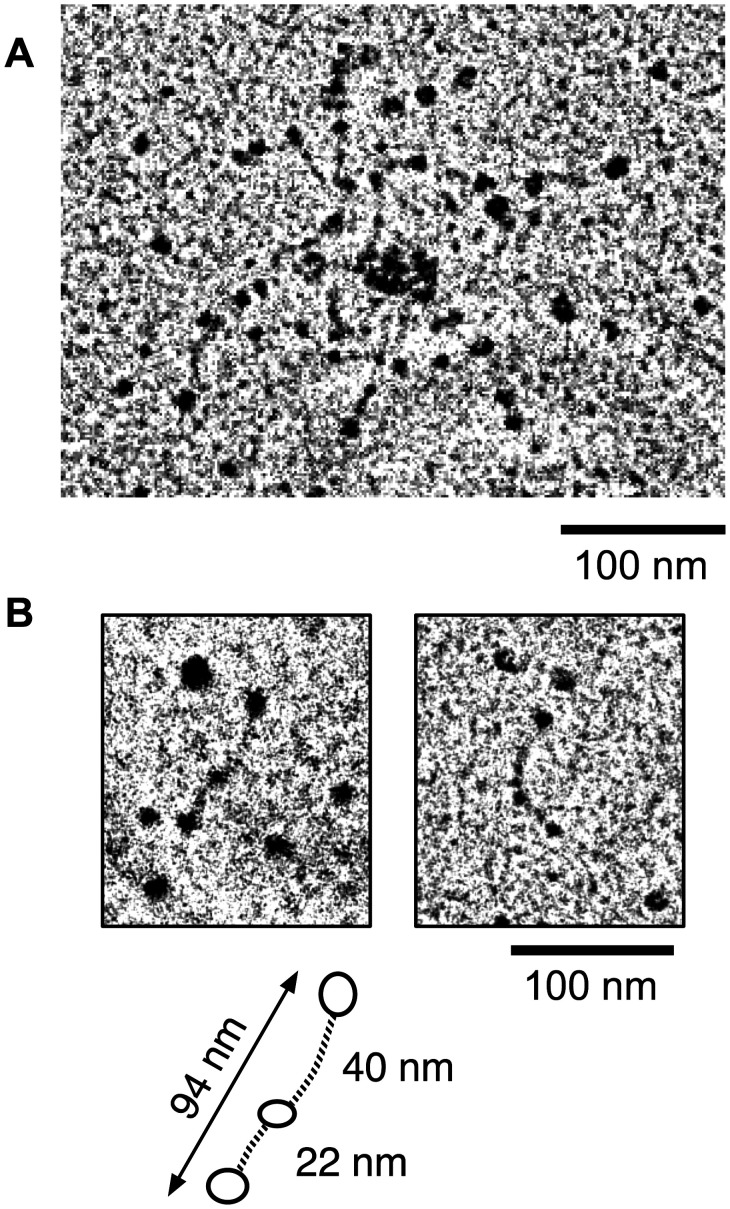
Rotary shadowing electron microscopy of *r*EPclB. (**A**) Internal structure of a small aggregate of *r*EPclB molecules. The micrograph suggests multiple flexible molecules, reminiscent of those observed for *r*EPclA, with dark globular structures (presumably globular domains of PfN, PfC and Pf2), and poorly defined linear structures (presumably stalks containing the PCoil and Col domains). The *r*EPclB molecules seem to aggregate heavily through one of the globular domains. (**B**) Possible examples of individual *r*EPclB molecules observed, isolated from the large aggregates, in some electron micrographs. (**C**) Interpretation of the observed in terms of three globular domains (PfN, Pf2 and PfC) connected by two stalk regions. Approximate molecular dimensions are shown for comparison purposes with *r*EPclA.

To investigate a possible structural organization of these aggregates, a sample of IPTG-induced *r*EPclB was used for rotary shadowing electron microscopy. The sample contained exclusively high-molecular weight aggregates that appeared in the electron micrographs as large masses of protein that, nevertheless, appeared to have a relatively narrow size distribution (300–500 nm in diameter, data not shown). Close inspection of the smallest aggregates (probably at an early stage of formation) revealed an internal structure that could be reconciled with entangled, multiple flexible linear beaded molecules (Figure 11). In the vicinity of these aggregates it was possible to discover individual features reminiscent of the *r*EPclA molecular morphology, but with three globular “domains” instead of two, connected by two flexible stalks (Figure 11). The terminal globular structures would correspond to the PfN and PfC domains, and the internal one would include the Pf2 domains. The flexible stalks would correspond to the two Col domains and the PCoil domain predicted in the *r*EPclB sequence. All these structures (and *r*EPclB) are presumed to be trimeric due to the presence of the PfC, Col and PCoil domains, all shown to trimerize in *r*EPclA. The structural organization for *r*EPclB would therefore be similar to that seen for *r*EPclA. However, an effective protocol to increase the amount of non-aggregated protein will be necessary to demonstrate these assumptions and to properly characterize the molecular architecture of *r*EPclB (work in progress).

**Figure 12:**
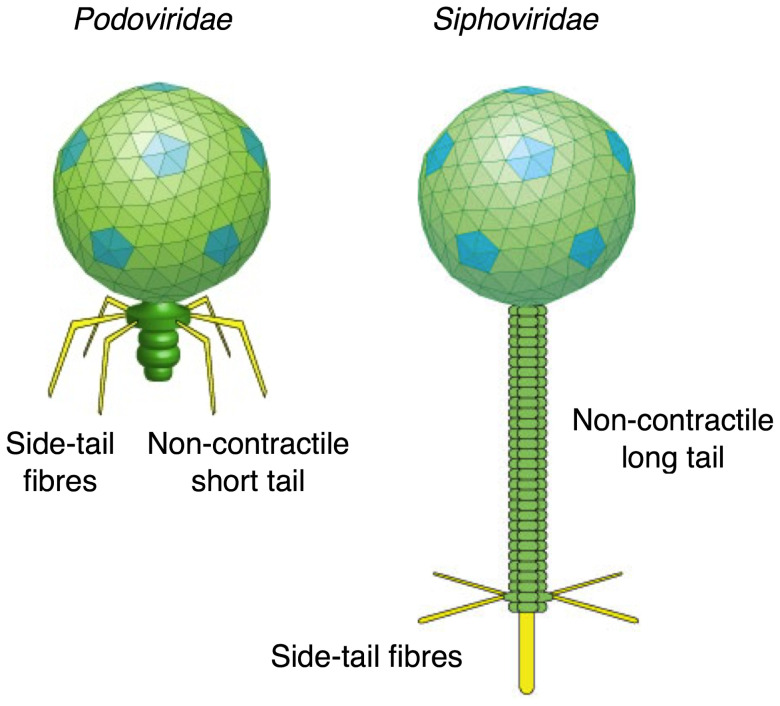
Typical morphologies of *podoviridae* and *siphoviridae* particles (reproduced with permission from ViralZone, Swiss Institute of Bioinformatics: www.expasy.org/viralzone**,** [**79**]**).** The representative 933W phage and most field isolates show a *podoviridae* morphology, with isometric capsids of about 60–70 nm in diameter and short tails of 10–30 nm in length [12], [56]. EPclPs would be the main components on the side tail fibres.

## Discussion

Multiple open reading frames with collagen-like amino acid sequences have been identified automatically in the genomes of several EHEC strains. These open reading frames are incorporated in the sequence regions of prophage and prophage-like elements embedded in these EHEC genomes (Tables 2 and 3) and presumably code for proteins involved in phage morphogenesis. Two recombinant proteins *r*EPclA and *r*EPclB, representative of the most common domain architectures EPclA and EPclB (Figure 1), were amplified from a sample of genomic DNA from the O157:H7 Sakai strain, cloned into appropriate protein expression vectors, and the resulting recombinant proteins analyzed biochemically. The aims were to demonstrate their biochemical viability and structural integrity, to confirm the presence of molecular characteristics typical of collagen-like proteins, to investigate their quaternary structure, conformation, morphology and thermal stability, and to analyze some of their individual domains.

Both *r*EPclA and *r*EPclB are produced as soluble proteins in *E. coli*, although *r*EPclB has a strong tendency to form large, soluble aggregates. Thus, most of the biochemical analysis has been done on *r*EPclA. Our data demonstrate that EPclA shows the main characteristics of collagen-like proteins: it forms stable trimers in solution that dissociate upon denaturation (Figure 3), and its collagen-like sequence adopts a collagen triple helical conformation, as demonstrated by CD spectroscopy (Figure 4). These data confirm that the collagen-like sequence (Col) of EPclA is a true collagen domain, and strongly suggests that collagen-like sequences in other EPclPs will adopt the same conformation.

The molecular morphology of *r*EPclA has been visualized by rotary shadowing electron microscopy. *r*EPclA is a trimeric dumbbell-shaped molecule, with two globular domains joined by a semi-flexible “stalk”, or rod-shaped domain (Figures 8–9). This connecting stalk is made in fact of two triple-helical domains: the collagen triple-helical domain (Col) and a trimeric α-helical coiled coil (PCoil) encompassing the region between the Col domain and the N-terminal PfN domains. The CD spectrum of *r*EPclA (Figure 5) is largely dominated by the combination of CD spectra from an α-helical coiled coil and a collagen triple helix. Coiled-coil prediction algorithms give high scores to the last 30 amino acids of the PfN domain and to the region between the PfN and Col domains, both for EPclA and EPclB (Figure 2). Our data indicates that, at least in EPclA, the region between PfN and Col forms indeed a trimeric α-helical coiled coil, as demonstrated by SEC/MALLS (Figure S3) and CD spectroscopy (Figure 6). We predict that similar PCoil domains in EPclB and other EPclPs will also form trimeric α-helical coiled coils. The combination of collagen triple helices adjacent to trimeric coiled coils is not unusual and there are many proteins for which such structural arrangement is predicted [45], [46], where the α-helical coiled coil functions mainly as an oligomerization domain.

The thermal stability of the Col domain of EPclA is higher than expected when compared to eukaryotic collagens, especially after considering the lack of prolyl hydroxylation (discussed below). The thermal denaturation data of *r*EPclA (Figures 4, 5, 6, 7) shows two sharp transitions, a first one at 42°C and a second one at 52°C (Figure 5). These two transitions correspond respectively to the loss of the collagen triple helix and the loss of the α-helical coiled coil. The sharpness of both melting curves indicates highly cooperative transitions for each case (including local chain dissociation as a result of conformational change). Nevertheless, EPclA will remain trimeric at temperatures between the melting of its Col (collagen triple helical) and PCoil (α-helical) domains. Identical transition temperatures are observed separately in two fragments: Col–PfC shows a single transition at 42°C, and PfN–PCoil shows a single transition at around 50°C, both consistent with the loss of conformation of their helical domains. The coincidence in transition temperatures with those of full-length *r*EPclA suggests that the two transitions are essentially independent of each other, and that the presence or absence of either the Col or PCoil domains does not change the thermal stability of the other one.

The thermal stability of the Col domain of EPclA appears remarkable. To remain functional at body temperatures, mammalian collagens have a high proportion of imino acids in their collagen domains (>20% in humans) and require a complicated mechanism of post-translational hydroxylation of proline residues [47], [48], [49]. The enzymes required for prolyl hydroxylation are not present in *E. coli* and yet, the melting temperature of EPclA Col domain (42°C) is higher than that of the much longer mammalian collagens (∼37°C, around body temperature [50]). Glycosylation of threonine residues, another mechanism of collagen stabilisation [40], is not observed in *r*EPclA either. Thus, the higher than expected thermal stability of the Col domain of EPclA could be the consequence of an unusually high proportion of Pro residues in the X position (Table 4), a high proportion of charged residues Arg, Asp, Glu and Lys (Table 4), and possibly a stabilizing effect by the PfC domains (see below). A stabilization mechanism based on a high proportion of charged residues has been proposed for the collagen triple helical domain of *S. pyogenes* collagen-like protein Scl2 [42], but the overall proportion of charged amino acids in the collagen domain of Scl2 (30%) is higher than the overall proportion of charged amino acids in the collagen domains of EPclPs (22%).

The thermal stability of the PCoil domain in EPclA is even higher. It is currently unknown if these high transition temperatures for the Col and PCoil domains have any functional significance. As EPclPs are likely to participate in phage morphogenesis, such high thermal stability may be required to ensure appropriate assembly of the phage particles during prophage induction from EHEC, while inside the host intestine, or to facilitate survival of free phages in the extraintestinal environment.

Electron micrographs of *r*EPclA show a degree of variable bending in the connecting stalk (Figure 9) that suggests the presence of a “molecular hinge” between the PCoil and Col domains. This hinge could be simply an area of structural discontinuity and increased flexibility between the collagen triple helix and the three-stranded α-helical coiled coil. Some discontinuity is expected: the transition between the two helical domains needs to account for a change in axial chain staggering, from three chains in register in the α-helical coiled coil to the one-residue stagger characteristic of the collagen triple helix [45]. It is also likely that the PCoil to Col transition changes its superhelical handedness, from a right-handed collagen superhelix to the most common left-handed coiled-coil superhelix. Although right-handed coiled coils have been described, they show undecad (11) or pentadecad (15) residue periodicity instead of the canonical heptad (7) periodicity of left-handed coiled coils [51], [52]. The sequence of the PCoil domain is unusual for an α-helical coiled coil, almost devoid of hydrophobic residues like leucine or isoleucine and with a high proportion of Ala and Ser residues (Figure 1). Thus, visualization of a clear repeating periodicity is difficult. Nevertheless, most of the PCoil domain and the last 30 residues of the PfN domain conform loosely to a seven-residue Ala-X-X-Ala/Ser-X-X-Ser periodicity that is given a high score by coiled-coil prediction algorithms. Additionally, differences in length between PCoil domains are multiples of seven (Tables 2 and 3), also consistent with a heptad repeat. Such periodicity would favour a left-handed superhelix for the PCoil domain. Whatever its structural details, the existence of a hinge or flexible discontinuity between the PCoil and Col domains is consistent with the independent thermal transitions observed in these two domains.

The globular domains of the *r*EPclA dumbbell are made of trimers of PfN and PfC domains. Analysis of the recombinant fragments PfN, PfN–PCoil, Trx–PfC and PfC show that PfC is a trimerization domain whereas PfN on its own is largely monomeric (Table 5). CD analysis of the PfN and PfN–PCoil fragments (Figure 8) shows that the PCoil domain is largely responsible for the α-helical features of the PfN–PCoil spectrum. The PfN spectrum also suggests some α-helical content in PfN domains (mostly predicted on the last 30 residues) that does not seem sufficient to form a three-stranded coiled-coil structure when the PCoil domain is not present. The CD spectrum of Col–PfC is entirely consistent with that of a collagen triple helix and indicates that the PfC domain has little or no α-helical structure.

The rapid reversibility of Col–PfC thermal denaturation (Figure 4) suggests that either the PfC domain remains stable and folded over the range of temperature used (4–55°C), or it unfolds at the same time as the Col domain but is able to refold rapidly when the temperature decreases. In either case the PfC domain is able to nucleate back the refolding of the triple helix in the Col domain. A similar behaviour has been observed with the N-terminal globular domain of the *S. pyogenes* collagen-like protein Scl2 [42]. The thermal denaturation of the PfN–PCoil fragment is partially reversible, and it appears that only the α-helical coiled-coil structure of the PCoil domain is quickly regained upon cooling (Figure 9). Thus, both the PfC and PCoil domains can be considered trimerization modules in EPclPs, and in the case of PfC, it may contribute in part to the high melting temperature of the Col domain.

Recent studies on collagen-like proteins of some pathogenic bacteria have revealed a variety of functions for these proteins, including binding extracellular matrix proteins or adherence to mammalian cells [25], [27], [28]. It is not currently known if EPclPs have any functional role for *E. coli* itself, or they are strictly phage morphogenetic proteins. Currently available evidence of EPclP expression in EHEC seems to be linked to phage induction [29], [30], [34], and the presence of common domains in sequences of EPclPs and side tail fibre proteins of lambda phages is a strong indicator of EPclPs as structural proteins in tail fibres from phages. The high thermal stability of EPclPs is also consistent with such a role.

All prophages and phages containing EPclP sequences are described as *Caudovirales*, or tailed bacteriophages. Bacteriophage 933W, isolated from the O157:H7 strain EDL933, was amongst the first EPclP-containing phages to be studied [53], [54], [55], [56]. Particles of 933W observed under the electron microscope show regular hexagonal heads (probably icosahedral), about 70 nm wide, and short tails 22–28 nm long and 13–17 nm wide [55], [56]. Often, 933W virions clump together through some form of tail-tail interaction [56]. Phage induction from EHEC strains results in virion particles with different morphologies [12], [34], [57], [58], that are usually classified as members of the *Podoviridae* or *Siphoviridae* families, with short or long non-contractile tails, respectively. Phages of these families often show tail fibres extending laterally from the sides of the tails (Figure 12), although none of the published electron micrographs of phages from EHEC strains has sufficient detail for their visualization. Tail fibres are often used for the phage particles to attach to the target cells, and this attachment triggers further events leading to injection of the viral DNA in the host cell periplasm.

The presence of PfN and Pf2 domains both in EPclP sequences and in side tail fibre proteins of λ bacteriophages [35], [36] strongly suggests (although do not absolutely prove) that EPclPs are major components of prophage side tail fibres and probably participate in adhesion to the *E. coli* cell surface, either directly or through assembly with other prophage tail fibre proteins. At least one instance of direct interaction has been proposed, between EPclB proteins of short-tailed Shiga toxin-carrying phages and the conserved *E. coli* protein YaeT. The study, which refers to EPclB as tail-spike protein, concludes that YaeT is the surface molecule recognized by the majority of these phages [59].

The use of the collagen triple helix by bacteriophages to build trimeric fibrillar proteins is remarkable. Trimerization is a highly prevalent characteristic of bacteriophage tail proteins and adhesins [60], [61] and novel trimeric folds have been discovered in bacteriophage fibre proteins [62], [63], [64], [65]. The α-helical coiled coil is present in bacteriophage fibre proteins such as fibritin [66] and our data confirms that phages have added collagen helices to their armoury of trimeric folds, in combination with α-helical coiled coils and other capping domains like PfN and PfC, the latest being a trimerization module itself. Furthermore, similarity with other viral fibrous tail proteins would suggest that the PfN domain contains the viral attachment site and the PfC domain is involved in binding to *E. coli* cell surface proteins [63]. The modular nature of EPclPs and other side tail fibre proteins, with different combinations of the same domains present in both closely and distantly related genomes, is consistent with a degree of recombination between multiple prophages on the same bacterial genome that often results in novel phages with an expanded host range and in new bacterial strains [34]. Prophage recombination and the efficiency of bacteriophages as HGT vehicles are responsible in part of the heterogeneity amongst EHEC strains and their rapid evolution. Thus, understanding the mechanisms of phage morphogenesis and phage interaction with the *E. coli* or EHEC cell surfaces may be more important than previously thought and deserve further investigation.

## Materials and Methods

### Sequence Retrieval and Analysis

Sequences of prophage collagen-like proteins from EHEC strains (EPclPs) were retrieved from the UniProt database [67]. These sequences were classified into different domain architectures (EPclA, EPclB, EPclC and EPclD, Figure 1) that were defined according to the occurrence and relative location of several conserved non-collagenous domains described in the InterPro [68] and Pfam databases [69] (Table 1). The probability of coiled-coil conformation and its oligomerization state in EPclP sequences were calculated with different prediction algorithms: *PCoils* [70], *Marcoil* [71], *MultiCoil* [72] and *SCORER 2.0* [73]. Secondary structure predictions for EPclP sequences were obtained from the *Jpred3* prediction server [74]. Default settings were used for all prediction algorithms.

### Cloning of EPclA and EPclB Sequences from E. coli O157:H7

Recombinant EPclA and EPclB proteins were produced using laboratory *E. coli* strains. Two DNA fragments coding for EPclA and EPclB were amplified from a sample of genomic DNA of *Escherichia coli* O157:H7 strain RIMD 0509952 (Sakai), which was a gift from Charles W. Penn (School of Biosciences, University of Birmingham, UK). Forward and reverse primers were designed from the nucleotide sequences of the open reading frames ECs2717 (EPclA) and ECs1228 (EPclB) of the *E. coli* Sakai strain genome [5], with accession numbers Q7ACX5 and Q8XAX7 (Uniprot), or NP_310744 and NP_309255 (NCBI), respectively. Appropriate *Nde* I and *Xho* I restriction sites were incorporated into the primer designs, with the resulting oligonucleotide sequences 5′-CAT ATG ATG GCA GTA AAG ATT TCA GGT GTA CTG-3′ (EpclA forward), 5′-CTC GAG TTC TCC TGT TCT GCC TGT ATC ACT GCC-3′ (EpclA reverse), 5′-CAT ATG ATG ACG ATG GAT CCG GGG GAG TAT GCG-3′ (EPclB forward), and 5′-CTC GAG TCA TTC TCC TGT TCT GCC TGT ATC ACT -3′ (EPclB reverse). The ECs2717 sequence was chosen amongst the six EPclA open reading frames from the Sakai genome as it had a putative promoter sequence (TGTTATGAC) 38 nucleotides upstream of the predicted start of the coding sequence. The products of PCR amplification were ligated into the pET-28a(+) expression vector (Novagen), and the correct frame and ligation of the EPclA and EPclB clones were confirmed by sequencing. The recombinant EPclA construct (*r*EPclA) was designed with hexahistidine tags both at the N- and C-terminus, whereas the recombinant EPclB construct (*r*EPclB) was designed with only an N-terminal hexahistidine tag (Figure S4). The entire nucleotide sequence for the *r*EPclA fragment could be obtained from the sequencing data and it was shown to contain twelve changes with respect to the closest deposited sequence (ECs2717). These changes resulted in four changes in the amino acid sequence (Figure S5). Each of these amino acid changes is a common substitution in other EPclA sequences from the Sakai and other O157:H7 strains. Thus, these changes correspond to normal sequence variability amongst EPclA proteins and are not artefacts introduced during PCR amplification.

### Cloning of PfN, PfN–PCoil, and PfC Fragments from EPclA

Separate recombinant constructs were prepared for three fragments of EPclA containing different predicted domains: PfN (residues 1-140), PfN–PCoil (residues 1-250) and PfC (residues 363-426) (Figure S4). All three fragments were amplified by PCR from the *r*EPclA clone using designed forward and reverse primers containing appropriate restriction sites: *Nhe* I and *Xho* I (PfN and PfN–PCoil) or *BamH* I and *EcoR* I (PfC). Sequences used for the different primers were 5′-CTC GTC GCT AGC ATG GCA GTA AAG ATT TCA-3′ (PfN and PfN–PCoil forward), 5′-CTC GTC CTC GAG TCA CTG ACT GGC TGA-3′ (PfN reverse), 5′-CTC GTC CTC GAG TCA CAC CAC GGT GGG-3′ (PfN–PCoil reverse), 5′-CTC GTC GGA TCC ATC CGT TTT CGT CTG GGGC-3′ (PfC forward), and 5′-CTC GTC GAA TTC CTA ATC CAG CCC CTT AAC ATC-3′ (PfC reverse). The products of PCR amplification were ligated into the pET-28a(+) expression vector (Novagen) and the correct frame and ligation of the clones were confirmed by sequencing. The PfC construct failed to produce any detectable expression and the PfC sequence was subsequently cloned into the fusion protein expression vector pHisTrx, an in house derivative of pET-32a (Novagen) encoding *E. coli* thioredoxin (*trx*) with an N-terminal hexahistidine tag and a thrombin cleavage site, followed by a unique multiple cloning site [75] (Figure S4).

### Expression and Purification of Recombinant Proteins

Recombinant proteins and fragments were produced in *E. coli* BL21(DE3) cells (*r*EPclA and *r*EPclB) or JM109(DE3) cells (PfN–PCoil, PfN and Trx–PfC), using both IPTG induction (small-scale test expression and large-scale expression) and auto-induction (only large-scale expression) [76]. Best expression conditions for IPTG induction were achieved by inoculating 5 ml cultures with single bacterial colonies expressing the recombinant proteins, followed by incubation at 37°C overnight, and then inoculating 500 ml cultures in 2-litre flasks with 1% overnight pre-culture, followed by incubation at 37°C until log phase, and induction with 1 mM IPTG followed by further incubation for 4 hours at 30°C. Best expression using auto-induction was achieved by inoculation with 1% overnight pre-culture of 500 ml cultures supplemented with auto-induction solutions (1 M each of Na_2_HPO_4_, KH_2_PO_4_, NH_4_Cl, Na_2_SO_4_ and MgSO_4_, 20 mM CaCl_2_, 50% glycerol, 1 M glucose and 20% lactose) followed by incubation overnight at 37°C. All recombinant products mainly localised to the soluble fraction, auto-induction expression being more effective in producing larger amounts of soluble protein. Recombinant proteins were purified by nickel-affinity chromatography (QIAGEN) using a 5 ml column and following the manufacturer protocols, followed by size-exclusion chromatography using a 120-ml HiLoad 16/60 Superdex 200 column (GE Healthcare), with 10 mM Tris, 150 mM NaCl, pH 7.4 as elution buffer. SDS-PAGE analysis showed that the bands corresponding to *r*EPclA and *r*EPclB migrated with apparent molecular weights of ∼66 kDa and ∼100 kDa, respectively (Figure S2 and data not shown), which are higher than their predicted molecular weights of 47 kDa and 66 kDa. Glycosylation was ruled out by a negative periodic acid-Schiff stain analysis (not shown), and thus the anomalous gel migration of purified *r*EPclA or *r*EPclB must relate to their collagen-like sequences, a behaviour commonly observed in collagens and collagen-like proteins. Overproduction of *r*EPclA by auto-induction produced significant amounts of two endogenous proteolytic fragments whose partial sequences were identified by peptide mass spectrometry (see below and Figure S2). A fragment containing only the Col and PfC domains from *r*EPclA (Col–PfC fragment, Figures S2 and S4) could be isolated in enough quantities from full-length *r*EPclA for subsequent biophysical characterisation. Conditions for consistent production of the two proteolytic fragments could not be established, and most samples of *r*EPclA produced by auto-induction and IPTG induction did show one major band in SDS-PAGE analyses of purified samples, corresponding to full length *r*EPclA (Figure S2A). In these cases, levels of the proteolytic fragments were too low for characterization and isolation.

### Gel electrophoresis, Immunoblot Analysis and Protein Identification

Protein samples were denatured by heating at 90°C for 10 minutes and electrophoretically separated in 0.1% SDS, 4–12% gradient NuPAGE Bis-Tris polyacrylamide gels (Invitrogen). Proteins were visualised via Coomassie Brilliant Blue staining, and their electrophoretic migrations compared to those of prestained molecular weight markers (Precision Plus All Blue standards, BioRad). All proteins and fragments containing the Col or PCoil domains showed a slower than normal electrophoretic migration and higher than expected apparent molecular weights in the gels. Thus, identities of individual protein bands had to be confirmed by in-gel trypsin digestion followed by reverse phase chromatography of the tryptic peptides and sequence identification with mass spectrometry in the Biomolecular Analysis Facility of the Faculty of Life Sciences, University of Manchester (BAF-FLS). Bands of interest were excised from the gels, reduced with 10 mM dithiothreitol and alkylated with 55 mm iodoacetamide. Samples were digested overnight with trypsin at 37°C and then analysed using a CapLC (Waters) nanoLC system coupled to a Q-TOF Micro Mass Spectrometer (Waters). Peptides were separated by reverse phase chromatography using a 0.075×150 mm PepMap column (Dionex) and an acetonitrile gradient in 0.1% formic acid. Peptides eluting from the column were selected automatically for fragmentation. Data were searched against the UniProt database using the *Mascot* engine (Matrix Science). Further confirmation by immunoblotting was performed via transfer of SDS-PAGE separated proteins onto nitrocellulose membranes (Whatman) followed by incubation with a commercial anti-His_6_ tag antibody. Horseradish peroxidase-coupled Fab-specific anti-mouse IgG (Sigma) was used as secondary antibody, and detection was achieved with SuperSignal West Pico Chemiluminescent Substrate (Thermo Scientific).

### Protein Concentration

Protein concentration was determined by measuring UV absorption at 280 nm with a NanoDrop ND-1000 spectrophotometer (Labtech International), using molar extinction coefficients calculated from the amino acid sequences of the different recombinant proteins and fragments [77]. For recombinant fragments without tryptophan residues in their sequences, a corrected value of the molar extinction coefficient was derived from the UV absorption at 280 nm of equal concentration samples in aqueous buffers and in 8 M urea. In these cases the extinction coefficient calculated from sequence was considered accurate enough for determination of the protein concentration in 8 M urea [77], and an adjusted extinction coefficient was derived for the protein in water-based buffers.

### Analytical Ultracentrifugation

Sedimentation equilibrium analysis of *r*EPclA was performed using an Optima XL-A ultracentrifuge (Beckman Instruments) from the BAF-FLS. The protein was in a 10 mM Tris, 150 mM NaCl, pH 7.4 buffer that was supplemented with increasing concentrations of guanidinium chloride (0–6 M). Sample volumes of 110 µl were used in six-sector Epon-filled centrepiece cells equipped with quartz windows. All experiments were conducted at 20°C and sedimentation equilibrium data were collected at 10,000, 18,000 and 28,000 rpm. Weight-averaged molar mass (*M_w_*) was determined using *Hetero* analysis (developed by J. Cole and J. Lary at the University of Connecticut), using a single ideal species model. A value of 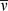 = 0.7139 was used for the partial specific volume of *r*EPclA, calculated from its amino acid sequence using *Sednterp*, version 1.09.

### Size Exclusion Chromatography and Multiangle Laser-Light Scattering

Molecular weights of the recombinant proteins were determined by size exclusion chromatography coupled to multiangle laser-light scattering (SEC/MALLS) in the BAF-FLS. Depending on their size, proteins were chromatographed in Superdex 200 10/300 GL (10 to 600 kDa), Superose 6 10/300 GL (5 to 5000 kDa), or Superdex 75 5/150 GL (3 to 70 kDa) columns, all from GE Healthcare, using 10 mM Tris pH 7.4, 150 mM NaCl, 1 mM EDTA as elution buffer. Elution from the column was continuously analysed in-line with a light scattering (LS) detector Dawn Heleos II (Wyatt Technology) and an Optilab rEX refractometer (Wyatt Technology). The LS intensity and eluant RI were analyzed using *ASTRA* software (version 5.21, Wyatt) to give a weight-averaged molar mass (*M_w_*). Fractions of 0.5 ml were collected for further analysis.

### Circular Dichroism (CD) Spectroscopy

Secondary structures and thermal denaturation of the recombinant proteins were analyzed by CD spectroscopy. Samples were equilibrated in 10 mM Tris, 150 mM NaCl, pH 7.4 and purified via size-exclusion chromatography before analysis. For the PfN–PCoil and PfN recombinant proteins a phosphate buffer with 20 mM Na_2_HPO_4_/NaH_2_PO_4_, 100 mM NaCl pH 7.4, was also used for CD analysis. CD spectra were recorded with a Jasco J-810 spectrometer equipped with a peltier temperature controller. Wavelength scans were performed using a 0.5 mm-path-length Starna quartz cell of acceptable birefringence for CD, and data were collected every 0.5 nm with a 1 nm bandwidth. Each spectrum was obtained from the accumulation of data from 10 scans; baseline was corrected using the spectrum of a Tris/NaCl buffer blank. Spectra were obtained at different temperatures, and thermal transition profiles were recorded between 4°C and 90°C at 220, 216 or 222 nm (depending on the protein being examined) with a data pitch of 0.5 nm, bandwidth of 1 nm, detector response time of 32 sec and temperature slope of 20°C/hr. Samples were cooled back to 4°C after the different transitions and final spectra were recorded at that temperature. Ellipticities in millidegrees were converted to mean residue molar ellipticities (degree cm^2^ dmol^-1^) by normalizing for the number of residues on each protein or fragment.

### Rotary Shadowing Electron Microscopy

The structural organization of the different recombinant proteins was investigated under an electron microscope after rotary shadowing using the mica sandwich technique [78]. Five µl of sample (5 µg/ml) were adsorbed onto freshly cleaved mica. Another freshly cleaved mica disc was then gently placed on top, causing the drop to spread evenly between the two mica discs. The sample was allowed to adsorb for 1 min, after which it was washed with water and then freeze-dried at –80°C in a Cressington CFE-50 freeze-fracture apparatus (Cressington Scientific Instruments). The dried sample was maintained under vacuum and its temperature was dropped to –190°C. Rotary shadowing was then performed at an angle of 5° relative to the mica surface by electron-beam evaporation of platinum-carbon. The platinum film thickness in the plane of the substrate was measured by a quartz-crystal film-thickness monitor as 1.8 Å; this film was subsequently coated with a 7 nm carbon backing layer. Replicas were floated from mica on distilled water and placed on 400 mesh copper grids. Photomicrographs were taken with an FEI Tecnai Twin transmission electron microscope (FEI, Eindhoven, Netherlands) operated at 120 kV. Digital images (1024×1024 pixels) were recorded on a TVIPS F214 cooled CCD camera. Magnification was calibrated using a diffraction grating replica (2160 lines/min; Agar Scientific, Stansted, UK).

## Supporting Information

Figure S1 Analysis of *r*EPclA and its Col–PfC fragment by SEC/MALLS. **(A)** Chromatogram showing the elution of nickel-affinity purified *r*EPclA from a Superose 6 10/300 GL size exclusion column; the red trace corresponds to the light scattering detector and the green trace to the UV absorption detector, both in arbitrary units. Peak 1 corresponds to the void volume and contains high molecular aggregates; peak 2 corresponds to native *r*EPclA. **(B)** Molar mass distribution or native *r*EPclA (peak 2 in **A**) measured by light scattering. The blue trace corresponds to the refractive index detector (in arbitrary units) and the dashed black line shows the weight-average molecular mass for each slice, as measured by the light scattering detector. The molar mass distribution is consistent with trimeric *r*EPclA (Table 5). (**C**) Chromatogram showing the elution of a nickel-affinity purified auto-induction sample of *r*EPclA from a Superdex 200 10/300 GL size exclusion column (traces as in panel **A**). Peak 1 corresponds to the void volume and contains high molecular aggregates; peaks 2 and 3 show molar mass distributions consistent with trimeric *r*EPclA and trimeric Col–PfC fragment, respectively (Table 5); peak 4 is consistent with monomeric *r*EPclA. (**D**) Molar mass distribution of peak 3 from **C** (Col–PfC) re-chromatographed in the same Superdex 200 column. The blue trace corresponds to the refractive index detector (arbitrary units) and the dashed black line shows the weight-average molecular mass for each slice, as measured by the light scattering detector. The molar mass distribution is consistent with trimeric Col–PfC (Table 5).
(PDF)

Figure S2 Large-scale expression of *r*EPclA: SDS-PAGE analysis of the different fractions after purification by nickel affinity chromatography. Individual peptides identified by mass spectrometry on each protein band are shown in red against the original EPclA sequence. (**A**) Overexpression of *r*EPclA by IPTG induction. Lane 1: molecular weight markers; lane 2: flow-through; lanes 3–4: fractions eluted with 5 mM and 100 mM imidazole (washes); lanes 5–10: fractions eluted with 1 M imidazole. The overexpressed band of *r*EPclA, confirmed by mass spectrometry, shows an apparent molecular weight of ∼66 kDa (higher than the true molecular weight of 47 kDa). (**B**) Overexpression of *r*EPclA by auto-induction. Lane 1: molecular weight markers; lanes 2–10: fractions eluted with 500 mM imidazole. The *r*EPclA band runs at ∼66 kDa, also confirmed by mass spectrometry. Two additional protein bands were identified by mass spectrometry as endogenous proteolytic fragments of *r*EPclA fragments. The mapped peptides reveal the extent and domain composition of each fragment. The band corresponding to the Col–PfC fragment shows an apparent molecular weight of ∼30 kDa (higher than the predicted molecular weight of ∼21 kDa). Another band at ∼60 kDa seems to correspond to a fragment with a partial digestion of the PfN domain and including the PCoil–Col–PfC domains.
(PDF)

Figure S3 Analysis of PfN, PfN–PCoil and Trx–PfC fragments by SEC/MALLS. (**A**) Chromatogram showing the elution of nickel-affinity purified PfN–PCoil fragment (green trace) or PfN fragment (red trace), from a Superdex 200 10/300 GL size exclusion column. Both traces correspond to the UV absorption detector, in arbitrary units. The dashed green and red lines show weight-average molecular masses for each slice of peaks 1 to 4, as measured by the light scattering detector. Peaks 1 and 2 correspond to trimeric and monomeric PfN–PCoil fragment, respectively, whereas peaks 3 and 4 correspond to trimeric and monomeric PfN. Molar mass distributions on each peak are consistent with these oligomerization states (Table 5). The predominant species in the PfN–PCoil sample is the trimer (peak 1), but a small amount of monomer (peak 2) can be detected. For PfN the predominant species is the monomer (peak 4), but a small amount of trimer (peak 3) can be detected. Elution volumes appear to be non-linear between the two purifications as the PfN–PCoil monomer elutes at a lower volume than the PfN trimer. (**B**) Molar mass distribution or the Trx–PfC fragment, measured by light scattering. The blue trace corresponds to the refractive index detector (in arbitrary units) and the dashed black line shows the weight-average molecular mass for each slice, as measured by the light scattering detector. The molar mass distribution is consistent with trimeric Trx–PfC (Table 5).
(PDF)

Figure S4 Domain architecture of the different recombinant proteins and constructs used in this study. Key to domain labels: PfN, phage fibre N-terminal domain; PCoil, phage coil domain; Col, collagen domain; PfC, phage fibre C-terminal domain; H, hexahistidine tag; Trx, thioredoxin tag.
(PDF)

Figure S5 Nucleotide and amino acid sequences of *r*EPclA from DNA sequencing of the product amplified from a sample of genomic DNA from *E. coli* O157:H7 Sakai and cloned into a pET-28a(+) expression vector (see Methods). Sequence colour code: red, PfN domain; orange, PCoil domain; green, Col domain; blue, PfC domain; black, additional amino acids introduced by cloning to the protein expression vector, including N-terminal and C-terminal hexahistidine tags and a thrombin cleavage site preceding the PfN domain. Twelve nucleotide changes with respect to the most similar deposited EPclA sequence (ECs2717) are highlighted in yellow. Of those, eight are silent and four lead to changes in the amino acid sequence, also highlighted in yellow. All these amino acid changes correspond to normal sequence variability amongst EPclA sequences from different O157:H7 strains.
(PDF)
