# Supplementary figures and images for: Collagen-Like Proteins in Pathogenic E. coli Strains
Source: PLoS One. 2012 Jun 6;7(6):e37872. doi: 10.1371/journal.pone.0037872 (PMC3368898; doi:10.1371/journal.pone.0037872)

**A**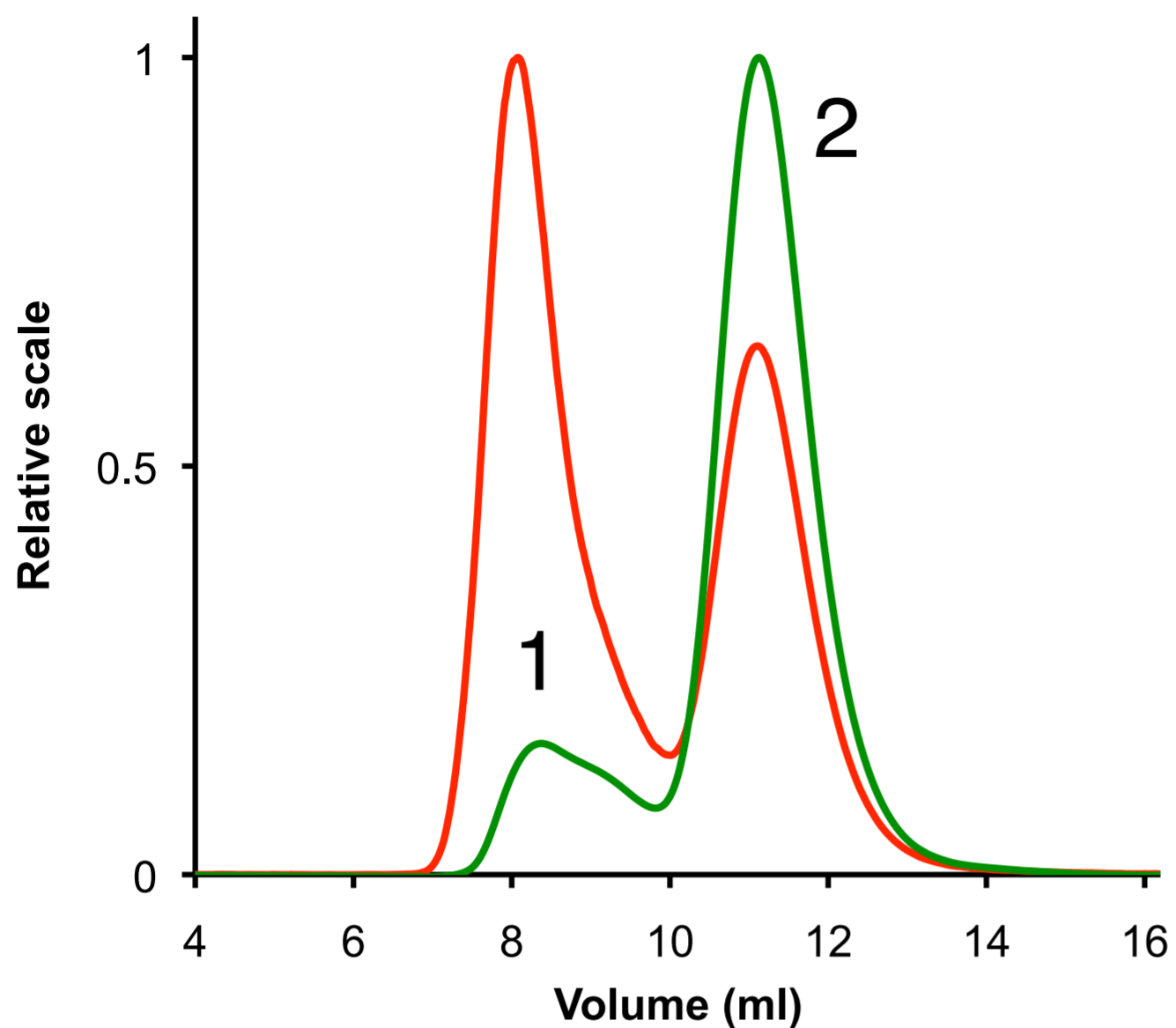**B**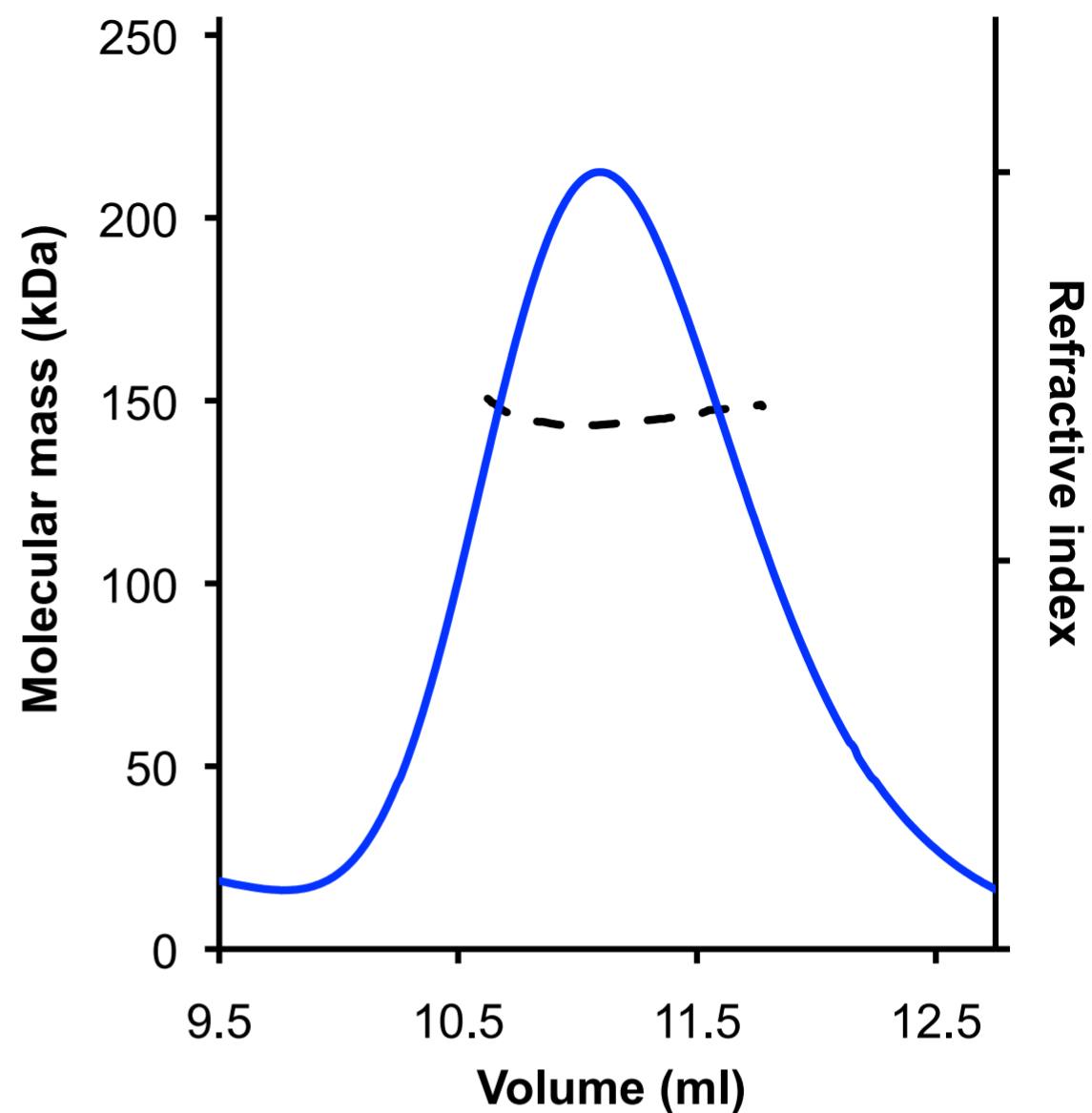**C**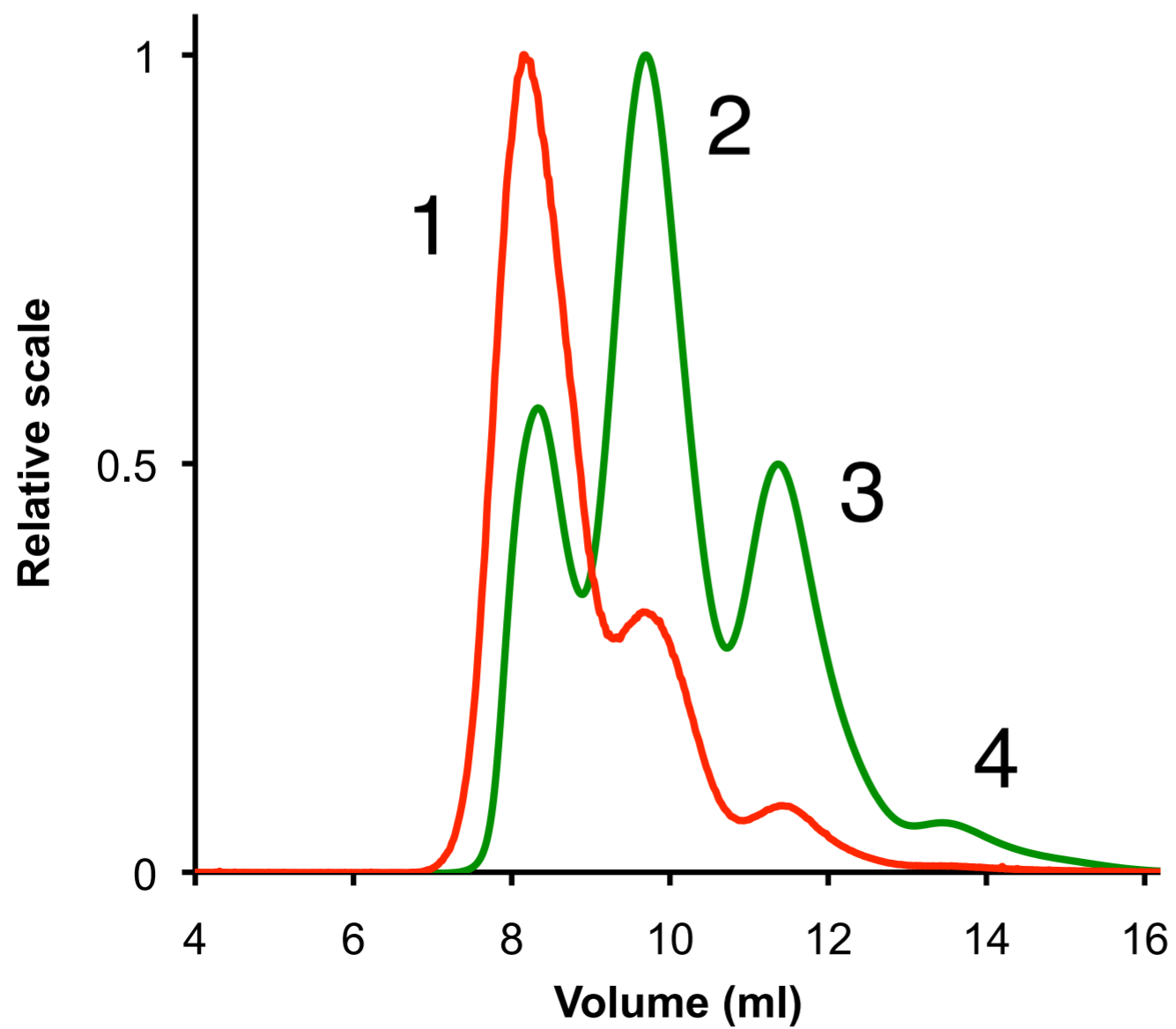**D**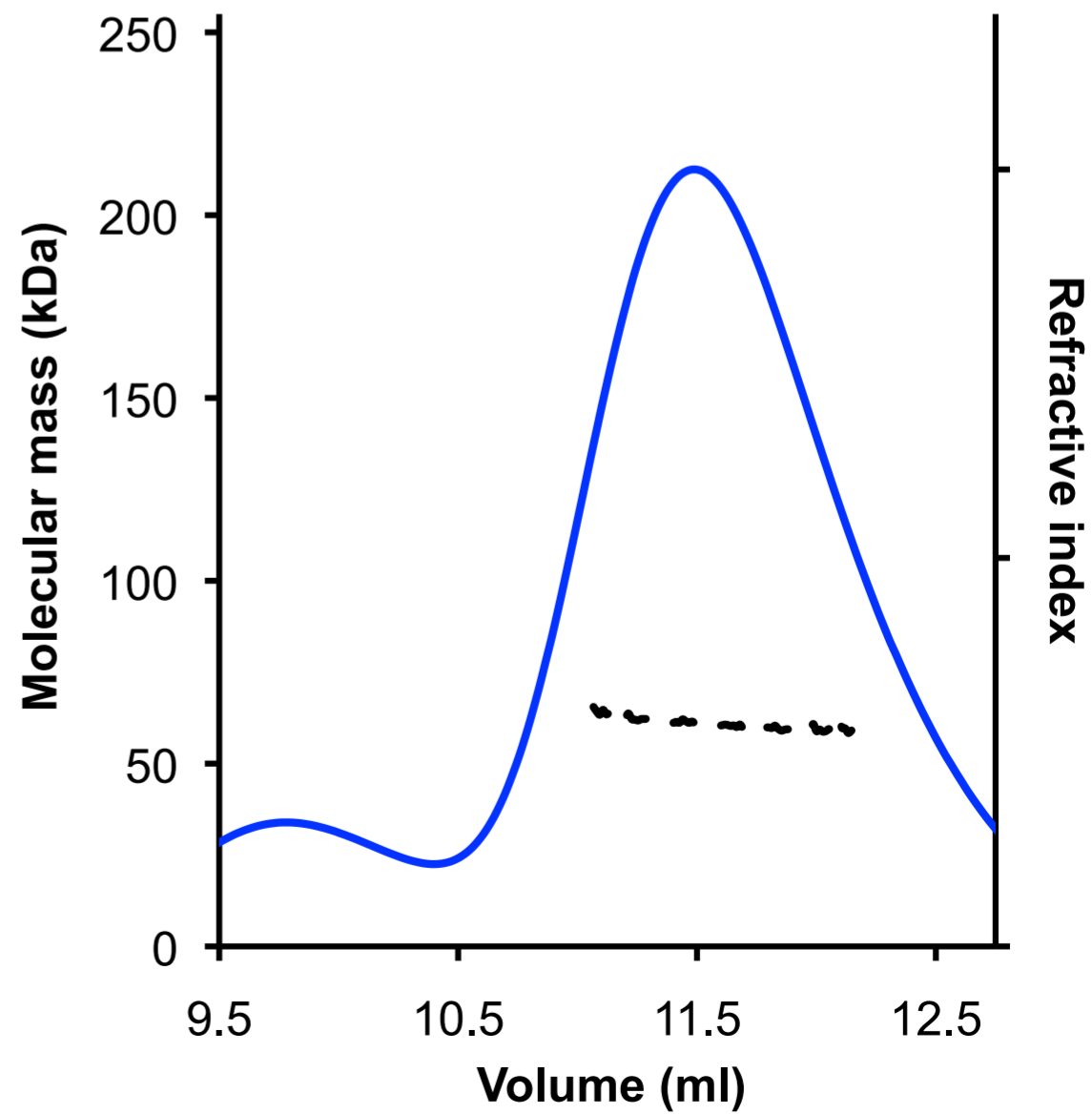

Supplement: Figure S1 — Analysis of rEPclA and its Col–PfC fragment by SEC/MALLS. (A) Chromatogram showing the elution of nickel-affinity purified rEPclA from a Superose 6 10/300 GL size exclusion column; the red trace corresponds to the light scattering detector and the green trace to the UV absorption detector, both in arbitrary units. Peak 1 corresponds to the void volume and contains high molecular aggregates; peak 2 corresponds to native rEPclA. (B) Molar mass distribution or native rEPclA (peak 2 in A) measured by light scattering. The blue trace corresponds to the refractive index detector (in arbitrary units) and the dashed black line shows the weight-average molecular mass for each slice, as measured by the light scattering detector. The molar mass distribution is consistent with trimeric rEPclA (Table 5). (C) Chromatogram showing the elution of a nickel-affinity purified auto-induction sample of rEPclA from a Superdex 200 10/300 GL size exclusion column (traces as in panel A). Peak 1 corresponds to the void volume and contains high molecular aggregates; peaks 2 and 3 show molar mass distributions consistent with trimeric rEPclA and trimeric Col–PfC fragment, respectively (Table 5); peak 4 is consistent with monomeric rEPclA. (D) Molar mass distribution of peak 3 from C (Col–PfC) re-chromatographed in the same Superdex 200 column. The blue trace corresponds to the refractive index detector (arbitrary units) and the dashed black line shows the weight-average molecular mass for each slice, as measured by the light scattering detector. The molar mass distribution is consistent with trimeric Col–PfC (Table 5). (PDF) [file pone.0037872.s001.pdf]

**A**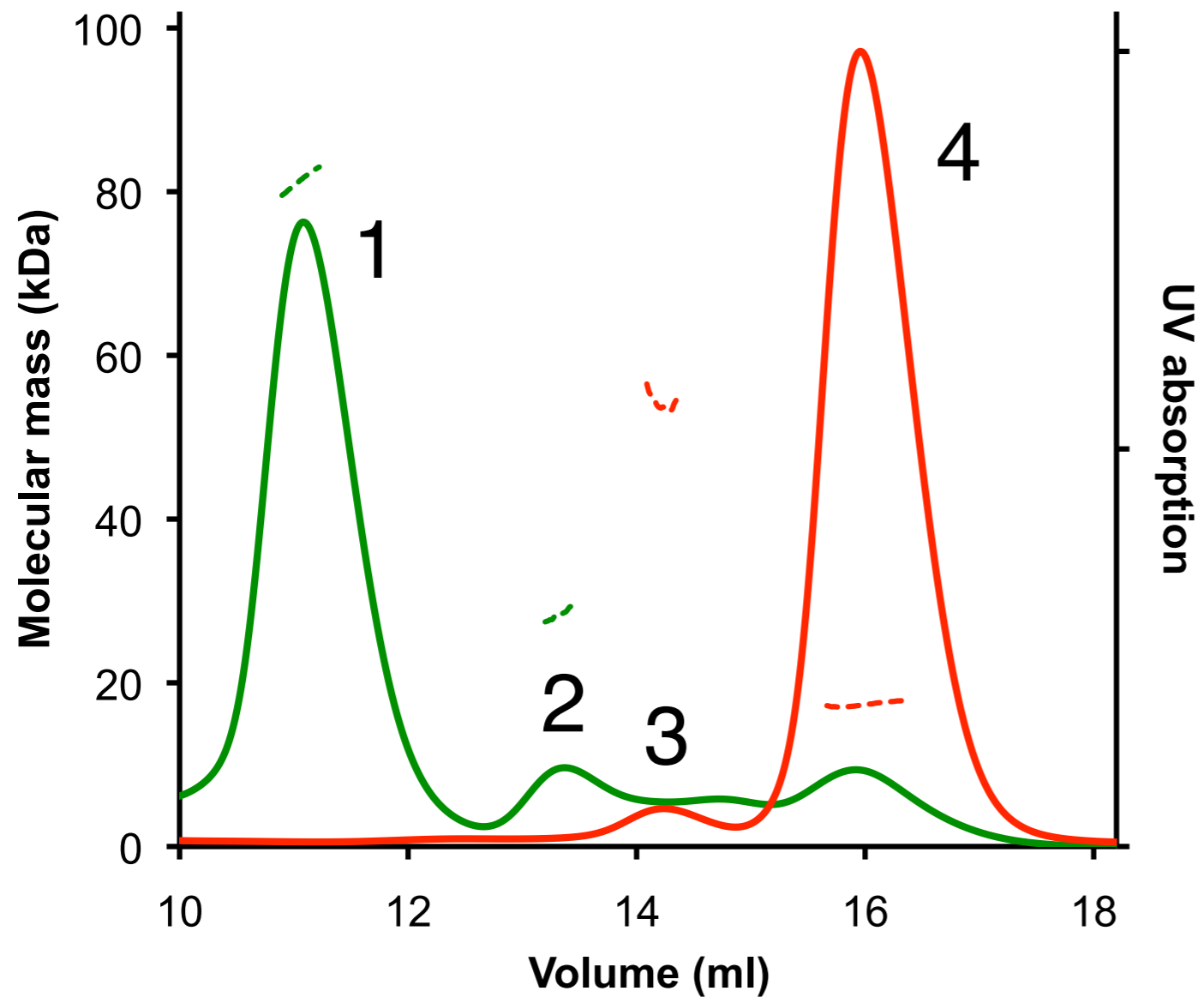**B**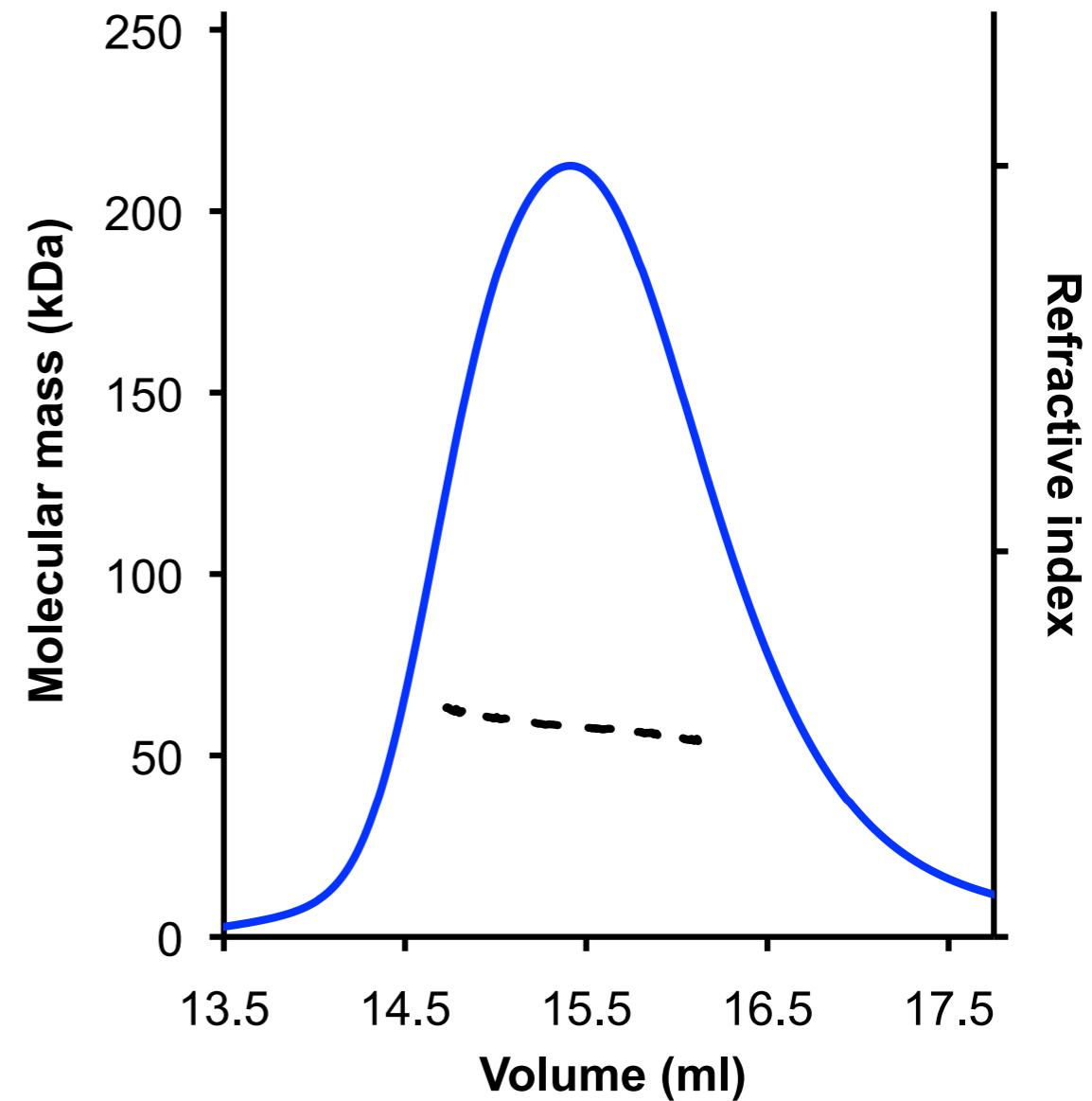

Supplement: Figure S3 — Analysis of PfN, PfN–PCoil and Trx–PfC fragments by SEC/MALLS. (A) Chromatogram showing the elution of nickel-affinity purified PfN–PCoil fragment (green trace) or PfN fragment (red trace), from a Superdex 200 10/300 GL size exclusion column. Both traces correspond to the UV absorption detector, in arbitrary units. The dashed green and red lines show weight-average molecular masses for each slice of peaks 1 to 4, as measured by the light scattering detector. Peaks 1 and 2 correspond to trimeric and monomeric PfN–PCoil fragment, respectively, whereas peaks 3 and 4 correspond to trimeric and monomeric PfN. Molar mass distributions on each peak are consistent with these oligomerization states (Table 5). The predominant species in the PfN–PCoil sample is the trimer (peak 1), but a small amount of monomer (peak 2) can be detected. For PfN the predominant species is the monomer (peak 4), but a small amount of trimer (peak 3) can be detected. Elution volumes appear to be non-linear between the two purifications as the PfN–PCoil monomer elutes at a lower volume than the PfN trimer. (B) Molar mass distribution or the Trx–PfC fragment, measured by light scattering. The blue trace corresponds to the refractive index detector (in arbitrary units) and the dashed black line shows the weight-average molecular mass for each slice, as measured by the light scattering detector. The molar mass distribution is consistent with trimeric Trx–PfC (Table 5). (PDF) [file pone.0037872.s003.pdf]

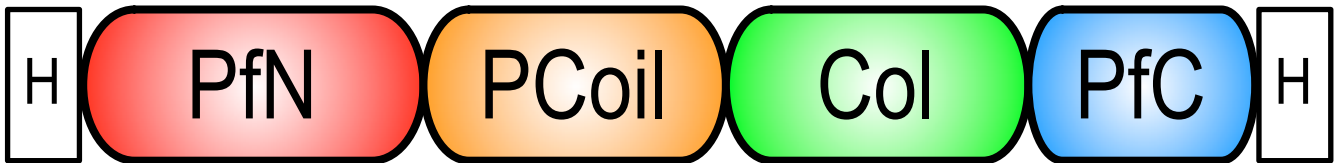

*rEPclA*

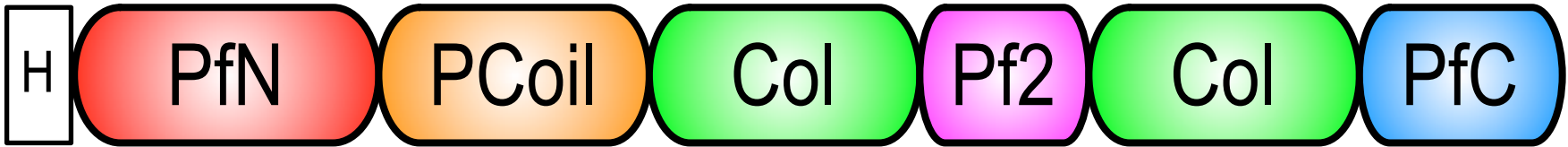

*rEPclB*

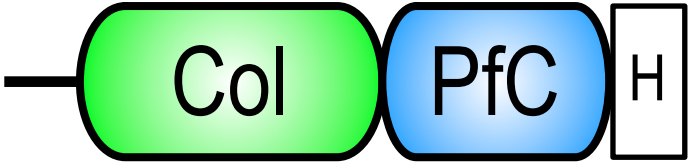

Col–PfC

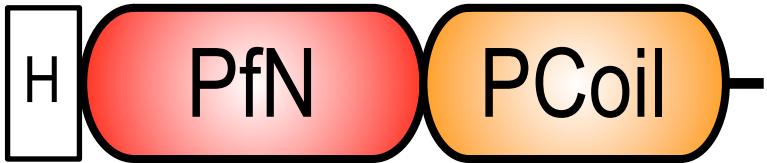

PfN–PCoil

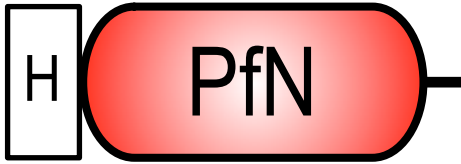

PfN

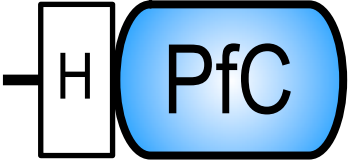

PfC

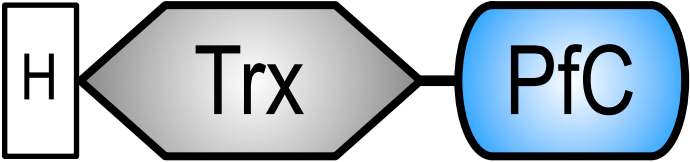

Trx–PfC

Supplement: Figure S4 — Domain architecture of the different recombinant proteins and constructs used in this study. Key to domain labels: PfN, phage fibre N-terminal domain; PCoil, phage coil domain; Col, collagen domain; PfC, phage fibre C-terminal domain; H, hexahistidine tag; Trx, thioredoxin tag. (PDF) [file pone.0037872.s004.pdf]
