# Supplementary material for: Collagen-Like Proteins in Pathogenic E. coli Strains
Source: PLoS One. 2012 Jun 6;7(6):e37872. doi: 10.1371/journal.pone.0037872 (PMC3368898; doi:10.1371/journal.pone.0037872)
Supplement: Figure S2 — Large-scale expression of rEPclA: SDS-PAGE analysis of the different fractions after purification by nickel affinity chromatography. Individual peptides identified by mass spectrometry on each protein band are shown in red against the original EPclA sequence. (A) Overexpression of rEPclA by IPTG induction. Lane 1: molecular weight markers; lane 2: flow-through; lanes 3–4: fractions eluted with 5 mM and 100 mM imidazole (washes); lanes 5–10: fractions eluted with 1 M imidazole. The overexpressed band of rEPclA, confirmed by mass spectrometry, shows an apparent molecular weight of ∼66 kDa (higher than the true molecular weight of 47 kDa). (B) Overexpression of rEPclA by auto-induction. Lane 1: molecular weight markers; lanes 2–10: fractions eluted with 500 mM imidazole. The rEPclA band runs at ∼66 kDa, also confirmed by mass spectrometry. Two additional protein bands were identified by mass spectrometry as endogenous proteolytic fragments of rEPclA fragments. The mapped peptides reveal the extent and domain composition of each fragment. The band corresponding to the Col–PfC fragment shows an apparent molecular weight of ∼30 kDa (higher than the predicted molecular weight of ∼21 kDa). Another band at ∼60 kDa seems to correspond to a fragment with a partial digestion of the PfN domain and including the PCoil–Col–PfC domains. (PDF) [file pone.0037872.s002.pdf]

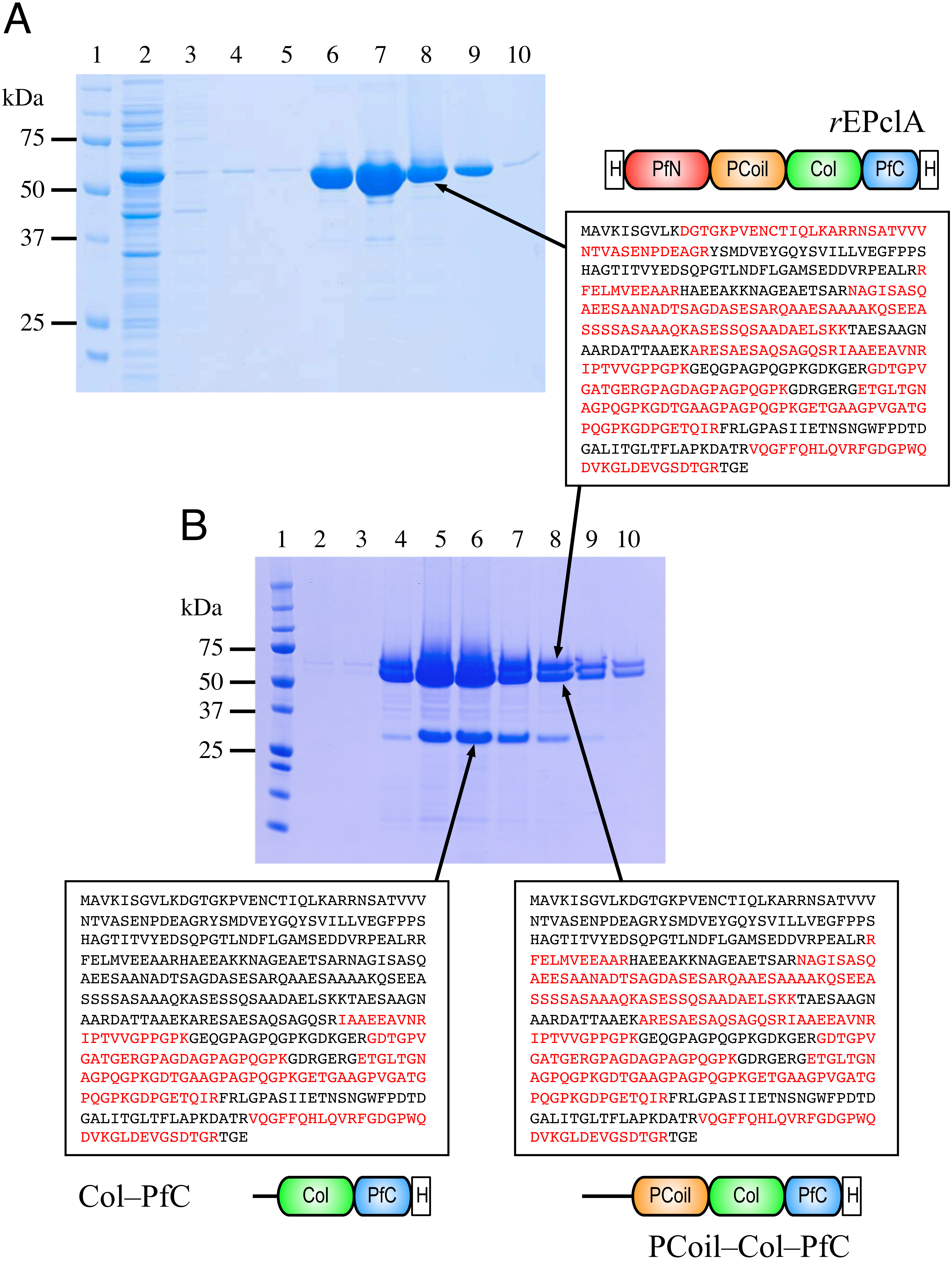
A
1 2 3 4 5 6 7 8 9 10
kDa
75
50
37
25
rEPclA
H PfN PCoil Col PfC H
MAVKISGVLKDGTGKPVENCTIQLKARRNSATVVV
NTVASENPDEAGRYSMDVEYGQYSVILLVEGFPPS
HAGTITVYEDSQPGTLNDFLGAMSEDDVRPEALRR
FELMVEEAARHAEEAKKNAGEAETSARNAGISASQ
AEESAANADTSAGDASESARQAAESAAAAKQSEEA
SSSSASAAAQKASESSQSAADAELSKKTAESAAGN
AARDATTAAEKARESAESAQSAGQSRIAAEEAVNR
IPTVVGPPGPKGEQGPAGPQGPKGDKGERGDTGPV
GATGERGPAGDAGPAGPQGPKGDRGERGETGLTGN
AGPQGPKGDTGAAGPAGPQGPKGETGAAGPVGATG
PQGPKGDPGETQIRFRLGPASIIETNSNGWFPDTD
GALITGLTFLAPKDATRVQGFFQHLQVRFGDGPWQ
DVKGLDEVGSDTGRTGE
B
1 2 3 4 5 6 7 8 9 10
kDa
75
50
37
25
MAVKISGVLKDGTGKPVENCTIQLKARRNSATVVV
NTVASENPDEAGRYSMDVEYGQYSVILLVEGFPPS
HAGTITVYEDSQPGTLNDFLGAMSEDDVRPEALRR
FELMVEEAARHAEEAKKNAGEAETSARNAGISASQ
AEESAANADTSAGDASESARQAAESAAAAKQSEEA
SSSSASAAAQKASESSQSAADAELSKKTAESAAGN
AARDATTAAEKARESAESAQSAGQSRIAAEEAVNR
IPTVVGPPGPKGEQGPAGPQGPKGDKGERGDTGPV
GATGERGPAGDAGPAGPQGPKGDRGERGETGLTGN
AGPQGPKGDTGAAGPAGPQGPKGETGAAGPVGATG
PQGPKGDPGETQIRFRLGPASIIETNSNGWFPDTD
GALITGLTFLAPKDATRVQGFFQHLQVRFGDGPWQ
DVKGLDEVGSDTGRTGE
MAVKISGVLKDGTGKPVENCTIQLKARRNSATVVV
NTVASENPDEAGRYSMDVEYGQYSVILLVEGFPPS
HAGTITVYEDSQPGTLNDFLGAMSEDDVRPEALRR
FELMVEEAARHAEEAKKNAGEAETSARNAGISASQ
AEESAANADTSAGDASESARQAAESAAAAKQSEEA
SSSSASAAAQKASESSQSAADAELSKKTAESAAGN
AARDATTAAEKARESAESAQSAGQSRIAAEEAVNR
IPTVVGPPGPKGEQGPAGPQGPKGDKGERGDTGPV
GATGERGPAGDAGPAGPQGPKGDRGERGETGLTGN
AGPQGPKGDTGAAGPAGPQGPKGETGAAGPVGATG
PQGPKGDPGETQIRFRLGPASIIETNSNGWFPDTD
GALITGLTFLAPKDATRVQGFFQHLQVRFGDGPWQ
DVKGLDEVGSDTGRTGE
Col–PfC
Col PfC H
PCoil Col PfC H
PCoil–Col–PfC
