# Supplementary material for: Collagen-Like Proteins in Pathogenic E. coli Strains
Source: PLoS One. 2012 Jun 6;7(6):e37872. doi: 10.1371/journal.pone.0037872 (PMC3368898; doi:10.1371/journal.pone.0037872)
Supplement: Figure S5 — Nucleotide and amino acid sequences of rEPclA from DNA sequencing of the product amplified from a sample of genomic DNA from E. coli O157:H7 Sakai and cloned into a pET-28a(+) expression vector (see Methods). Sequence colour code: red, PfN domain; orange, PCoil domain; green, Col domain; blue, PfC domain; black, additional amino acids introduced by cloning to the protein expression vector, including N-terminal and C-terminal hexahistidine tags and a thrombin cleavage site preceding the PfN domain. Twelve nucleotide changes with respect to the most similar deposited EPclA sequence (ECs2717) are highlighted in yellow. Of those, eight are silent and four lead to changes in the amino acid sequence, also highlighted in yellow. All these amino acid changes correspond to normal sequence variability amongst EPclA sequences from different O157:H7 strains. (PDF) [file pone.0037872.s005.pdf]

```
atgggcagcagccatcatcatcatcatcacagcagcggcctggtgccgcgcggcagccat
 M  G  S  S  H  H  H  H  H  H  S  S  G  L  V  P  R  G  S  H
atggcagtaaagatttcaggtgtactgaaagacggcacaggaaaaccggtagagaactgc
 M  A  V  K  I  S  G  V  L  K  D  G  T  G  K  P  V  E  N  C
accattcaactgaaagccagacgtaacagcgccacggtggtggtgaacacggtggcctct
 T  I  Q  L  K  A  R  R  N  S  A  T  V  V  V  N  T  V  A  S
gaaaatccggatgaagccggtcgttacagcatggacgttgagtacggtcagtacagcgtt
 E  N  P  D  E  A  G  R  Y  S  M  D  V  E  Y  G  Q  Y  S  V
attctgttggtggaagggttcccgccgtcacatgccggaccatcaccgtgtatgaagat
 I  L  L  V  E  G  F  P  P  S  H  A  G  T  I  T  V  Y  E  D
tctcaaccggggacgctgaatgatttctcggtgccatgtcggaggatgacgtccggccg
 S  Q  P  G  T  L  N  D  F  L  G  A  M  S  E  D  D  V  R  P
gaggcactgcgtcgttttgaactgatggtggaagaagcggcgcgtcacgctgaggaggcg
 E  A  L  R  R  F  E  L  M  V  E  E  A  A  R  H  A  E  E  A
aagaagaatgccggagaggcggagacgtccgcgaggaatgccggcatatcagccagtcag
 K  K  N  A  G  E  A  E  T  S  A  R  N  A  G  I  S  A  S  Q
gcagaagagagcgcggcaaatgctgacacttcagcaggggatgcatcggagtcagcccgg
 A  E  E  S  A  A  N  A  D  T  S  A  G  D  A  S  E  S  A  R
caggcggcagaaagtgcagccgctgcaaagcagtcagaggaggcgtcctcgtcctcggcc
 Q  A  A  E  S  A  A  A  A  K  Q  S  E  E  A  S  S  S  S  A
tctgcggccgctcaaaaagccagtgagtcatcacaaagtgcagcagatgctgagttgtca
 S  A  A  A  Q  K  A  S  E  S  S  Q  S  A  A  D  A  E  L  S
aaaaagacggcagaaagtgcagccggtaatgcagccagggatgcaacgaccgcaacagaa
 K  K  T  A  E  S  A  A  G  N  A  A  R  D  A  T  T  A  T  E
aaagcccgggagtcagcagaaagcgcacagtcagcggaacaaagcaggatagcggcggaa
 K  A  R  E  S  A  E  S  A  Q  S  A  E  Q  S  R  I  A  A  E
gaggccgtaaaccgaatccccaccgtggtgggacctcccggccaaaggggaacagggg
 E  A  V  N  R  I  P  T  V  V  G  P  P  G  P  K  G  E  Q  G
cccgcgggtcctcagggccgaagggtgataagggagagcgcggtgacaccggccctgtc
 P  A  G  P  Q  G  P  K  G  D  K  G  E  R  G  D  T  G  P  V
ggggcaaccggcgaacggggaccggcaggtgatgctggtccggcaggcccgcagggccg
 G  A  T  G  E  R  G  P  A  G  D  A  G  P  A  G  P  Q  G  P
aaaggtgacaggggagagcggggagagaccggtctgacgggaaatgcaggtccacagggt
 K  G  D  R  G  E  R  G  E  T  G  L  T  G  N  A  G  P  Q  G
ccaaagggagataccggtgcggcaggcccggcaggcccacagggaccgaaaggagaaaca
 P  K  G  D  T  G  A  A  G  P  A  G  P  Q  G  P  K  G  E  T
ggtgcggctggcccggtgggggcaaccggacctcagggaccgaagggcgacccgggggag
 G  A  A  G  P  V  G  A  T  G  P  Q  G  P  K  G  D  P  G  E
acacaaatccgttttcgtctggggccggcgagcattattgagacaaacagcaatggctgg
 T  Q  I  R  F  R  L  G  P  A  S  I  I  E  T  N  S  N  G  W
ttcccggatacagatggcgcactcatcaccggactgacctttcttgcccccaaagatgcc
 F  P  D  T  D  G  A  L  I  T  G  L  T  F  L  A  P  K  D  A
acacgggttcagggtttttttcagcatttgcaggtcaggtttggtgacgggccgtggcag
 T  R  V  Q  G  F  F  Q  H  L  Q  V  R  F  G  D  G  P  W  Q
gatgttaagggggctggatgaagtgggcagtgatacaggcagaacaggagaactcgagcac
 D  V  K  G  L  D  E  V  G  S  D  T  G  R  T  G  E  L  E  H
caccaccaccaccactga
 H  H  H  H  H  -
```
